# Epigenomic landscapes during prefrontal cortex development and aging in rhesus

**DOI:** 10.1093/nsr/nwae213

**Published:** 2024-06-18

**Authors:** Chao Ning, Xi Wu, Xudong Zhao, Zongyang Lu, Xuelong Yao, Tao Zhou, Lizhi Yi, Yaoyu Sun, Shuaishuai Wu, Zhenbo Liu, Xingxu Huang, Lei Gao, Jiang Liu

**Affiliations:** National Laboratory of Biomacromolecules, CAS Center for Excellence in Biomacromolecules, Institute of Biophysics, Chinese Academy of Sciences, Beijing 100101, China; College of Life Sciences, University of Chinese Academy of Sciences, Beijing 100049, China; National Laboratory of Biomacromolecules, CAS Center for Excellence in Biomacromolecules, Institute of Biophysics, Chinese Academy of Sciences, Beijing 100101, China; College of Life Sciences, University of Chinese Academy of Sciences, Beijing 100049, China; Division of HIV/AIDS and Sex-Transmitted Virus Vaccines, Institute for Biological Product Control, National Institutes for Food and Drug Control (NIFDC), State Key Laboratory of Drug Regulatory Science, Beijing 102629, China; Key Laboratory of Animal Models and Human Disease Mechanisms of Chinese Academy of Sciences, Kunming Institute of Zoology, Chinese Academy of Sciences, Kunming 650223, China; School of Life Science and Technology, Shanghai Tech University, Shanghai 201210, China; College of Life Sciences, University of Chinese Academy of Sciences, Beijing 100049, China; Guangzhou Nvwa Life Technology Co., Ltd, Guangzhou 510535, China; Shenzhen Neher Neural Plasticity Laboratory, CAS Key Laboratory of Brain Connectome and Manipulation, The Brain Cognition and Brain Disease Institute, Shenzhen Institute of Advanced Technology, Chinese Academy of Sciences, Shenzhen 518055, China; College of Life Sciences, University of Chinese Academy of Sciences, Beijing 100049, China; College of Life Sciences, University of Chinese Academy of Sciences, Beijing 100049, China; National Laboratory of Biomacromolecules, CAS Center for Excellence in Biomacromolecules, Institute of Biophysics, Chinese Academy of Sciences, Beijing 100101, China; College of Life Sciences, University of Chinese Academy of Sciences, Beijing 100049, China; Key Laboratory of Epigenetic Regulation and Intervention, Institute of Biophysics, Chinese Academy of Sciences, Beijing 100101, China; National Laboratory of Biomacromolecules, CAS Center for Excellence in Biomacromolecules, Institute of Biophysics, Chinese Academy of Sciences, Beijing 100101, China; Key Laboratory of Epigenetic Regulation and Intervention, Institute of Biophysics, Chinese Academy of Sciences, Beijing 100101, China; School of Life Science and Technology, Shanghai Tech University, Shanghai 201210, China; Zhejiang Provincial Key Laboratory of Pancreatic Disease, The First Affiliated Hospital, and Institute of Translational Medicine, Zhejiang University School of Medicine, Hangzhou 310058, China; National Laboratory of Biomacromolecules, CAS Center for Excellence in Biomacromolecules, Institute of Biophysics, Chinese Academy of Sciences, Beijing 100101, China; Key Laboratory of Epigenetic Regulation and Intervention, Institute of Biophysics, Chinese Academy of Sciences, Beijing 100101, China; National Laboratory of Biomacromolecules, CAS Center for Excellence in Biomacromolecules, Institute of Biophysics, Chinese Academy of Sciences, Beijing 100101, China; College of Life Sciences, University of Chinese Academy of Sciences, Beijing 100049, China; Key Laboratory of Epigenetic Regulation and Intervention, Institute of Biophysics, Chinese Academy of Sciences, Beijing 100101, China; Center for Excellence in Animal Evolution and Genetics, Chinese Academy of Sciences, Kunming 650223, China

**Keywords:** rhesus macaque, prefrontal cortex, 3D genomics, chromatin immunoprecipitation sequencing, development, aging

## Abstract

The prefrontal cortex (PFC) is essential for higher-level cognitive functions. How epigenetic dynamics participates in PFC development and aging is largely unknown. Here, we profiled epigenomic landscapes of rhesus monkey PFCs from prenatal to aging stages. The dynamics of chromatin states, including higher-order chromatin structure, chromatin interaction and histone modifications are coordinated to regulate stage-specific gene transcription, participating in distinct processes of neurodevelopment. Dramatic changes of epigenetic signals occur around the birth stage. Notably, genes involved in neuronal cell differentiation and layer specification are *pre*-configured by bivalent promoters. We identified a *cis*-regulatory module and the transcription factors (TFs) associated with basal radial glia development, which was associated with large brain size in primates. These TFs include GLI3, CREB5 and SOX9. Interestingly, the genes associated with the basal radial glia (bRG)-associated *cis*-element module, such as SRY and SOX9, are enriched in sex differentiation. Schizophrenia-associated single nucleotide polymorphisms are more enriched in super enhancers (SEs) than typical enhancers, suggesting that SEs play an important role in neural network wiring. A *cis*-regulatory element of *DBN1* is identified, which is critical for neuronal cell proliferation and synaptic neuron differentiation. Notably, the loss of distal chromatin interaction and H3K27me3 signal are hallmarks of PFC aging, which are associated with abnormal expression of aging-related genes and transposon activation, respectively. Collectively, our findings shed light on epigenetic mechanisms underlying primate brain development and aging.

## INTRODUCTION

The primate brain undergoes significant expansion in the neocortex and specialization of areas, particularly the prefrontal cortex (PFC), which serves as the anatomical basis for higher-order cognitive function, emotional regulation and behavioral complexity [1–4]. As a close evolutionary relative of humans [5], the rhesus monkey (*Macaca mulatta*) shares more similar patterns of gene regulation in the brain with humans than mice do [6]. Moreover, many neurological and neuropsychiatric diseases in humans are not adequately recapitulated in rodent models [7]. Thus, the rhesus monkey can serve as a valuable proxy for studying human brain development, although human brain development is still highly divergent, particularly in the PFC, with an extremely protracted timeline [1].

The process of brain development can be divided into four stages: neuronal fate determination and expansion, neuronal migration, synapse formation and neural network establishment. During embryonic stages, the generation of neural tissue (which occurs at the third gestational week in humans) begins with the induction of ectoderm into neuroectoderm, followed by the formation of the neural tube through neurulation [8]. Cortical parcellation can be explained by the Protomap Hypothesis (PMH), which suggests that the regional destiny of cortical neurons and the relative size of the cortical area are genetically determined early during embryonic development [9]. The expansion of the cortical surface during development begins with the increase in symmetrical divisions of neural stem cells in the ventricular zone (VZ). This is followed by the onset of neurogenesis and the formation of subventricular (SVZ), intermediate (IZ) and subplate (SPZ) zones, and cortical plate (CP) below the marginal zone (MZ) [9,10]. Based on the location of their mitosis, neural stem and progenitor cells fall into two groups: apical progenitors (APs) undergoing mitosis at the VZ, and basal progenitors (BPs) undergoing mitosis at the SVZ [11]. The BPs in the outer subventricular zone (OSVZ) consist of five types of precursors, including intermediate progenitor (IP) cells, basal process-bearing basal radial glial (bRG-basal-P) cells, apical and basal process-bearing bRG (bRG-both-P) cells, apical process-bearing bRG (bRG-apical-P), and transient bRG (tbRG) cells, which alternate between stages showing either an apical and/or a basal process and stages with no process. [12]. These basal radial glia (bRGs) cells in the OSVZ are considered the major cells contributing to neuronal expansion in primate brains [13–22]. Postmitotic neocortical excitatory neurons migrate radially along the glial fibers of mother radial glial (RG) cells in an inside-out manner, resulting in layer specification in the cortex [23–26] during the second trimester in humans. After reaching their specific cortical layers, the neurons undergo maturation and generate redundant synapses. The neurons then begin synaptic pruning and establish a connection with other neurons after birth until juvenile stage [26–28].

The transcriptional atlas of rhesus brains during development has been described [29,30]. However, the programs regulating gene expression during brain development remain incompletely understood. Epigenetic mechanisms play a pivotal role in the regulation of gene expression. Epigenetic regulation includes chromatin higher-order structure, chromatin accessibility, histone modifications, DNA methylation and so on. Topologically associating domains (TADs) are fundamental units of 3D nuclear organization at a sub-mega base scale, proposed to serve as structural scaffolds for the establishment of regulatory landscapes (RLs) [31]. CTCF is a key factor involved in chromatin structure, functioning as a chromatin insulator and mediating long-range chromatin interactions [32]. Accessible chromatin regions typically consist of *cis*-regulatory elements (CREs), such as promoters and enhancers, which can be bound by transcription factors (TFs) to activate gene expression [33–35]. Histone modifications mark distinct chromatin states. For example, H3K4me3 is associated with active promoters [36], H3K27ac is associated with active enhancers and promoters [37], and H3K27me3 is associated with a repressive chromatin state mediated by the Polycomb complex [38]. In addition, RNA polymerase II (Pol2) signal is associated with the RNA transcription [39]. DNA methylation in promoters can silence gene expression.

The epigenomic landscapes provide instructive information for annotating CREs and other non-exonic genomic regions for context-specific gene regulation. This study applied Hi-C, DNase I hypersensitive site sequencing (DNase-seq), chromatin immunoprecipitation sequencing (ChIP-seq), and RNA-seq technologies to profile a comprehensive CRE atlas of the rhesus PFC across prenatal, postnatal and aging stages. The landscapes of CREs enable us to illustrate the regulatory networks during PFC development, particularly during aging stages, which are largely uncharacterized. The multidimensional data obtained in this study are also valuable for interpreting how intergenic genome-wide association studies (GWAS) loci regulate gene expression and further contribute to disease susceptibility.

## RESULTS

### Charting epigenetic landscapes during rhesus macaque PFC development

**Figure 1.**
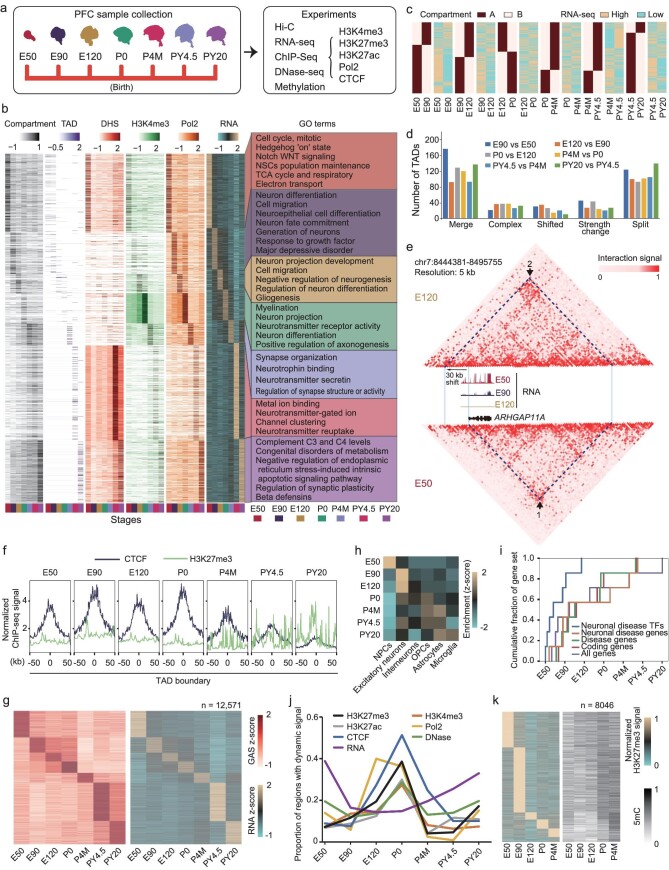
The epigenetic landscapes of the rhesus PFC during development and aging. (a) Experimental design for the study of epigenetic landscapes and transcriptome of the rhesus PFC during development and aging. E50, embryonic day 50; P4M, postnatal 4 months; PY4.5, postnatal 4.5 years. (b) Heat maps showing the normalized epigenetic signals and expression levels for the genes with stage-specific epigenetic signals or Pol2 binding signals during PFC development and aging. The enriched GO terms of genes in each cluster are shown on the right. (c) The expression states of the genes located in compartments undergoing compartment switch between adjacent developmental stages. (d) Bar plots showing the numbers of TADs with different types of TAD changes between adjacent developmental stages. (e) Heat map showing chromatin interaction signals at *ARHGAP11A* locus in rhesus PFC at the E50 and E120 stages. The RNA expression pattern of this gene is also shown. The interaction between TAD boundaries with signal changes are labeled by arrows. The TAD boundary is shifted to a locus 30 kb upstream from the E50 to E120 stage. (f) Metaplot showing ChIP-seq signals of CTCF and H3K27me3 across TAD boundaries in rhesus PFC at different stages. (g) Heat maps showing the gene active signals (GASs) and expression levels for the genes with stage-specific GASs. The number of the genes is shown. (h) Heat map showing the enrichment of genes with stage-specific GASs in the marker genes of major neural cell types. NPC, neural progenitor cell; OPC, oligodendrocyte precursor cell. (i) Cumulative fraction analysis of gene expression across different developmental stages. The gene sets consist of neuronal disease-associated TFs, neuronal disease-associated genes, all disease-associated genes, protein coding genes and all genes. (j) Line plots showing the proportion of epigenetic-signal-enriched regions (or expressed genes) undergoing significant signal (or expression) changes at the next stage. For the PY20 stage, the proportion of epigenetic-signal-enriched regions (or expressed genes) was calculated through comparison with the PY4.5 stage. (k) Heat map showing H3K27me3 signals and DNA methylation (5mC) levels of gene promoters exhibiting stage-specific H3K27me3 signals in rhesus PFCs.

Epigenetic information, such as higher-order chromatin structures, chromatin accessibility and histone modifications, has not been systematically charted during rhesus brain development. To understand the epigenetic mechanisms of PFC development in rhesus, PFC samples were collected at seven developmental stages, including embryonic day 50 (E50), E90, E120, postnatal day 0 (P0), postnatal four months (P4M), postnatal year 4.5 (PY4.5) and PY20, to profile the epigenetic landscapes (Fig. 1a, Table S1). These stages were selected to cover major lifespan time points, from prenatal to old stages, encompassing key developmental events of PFCs, including neuroepithelial progenitor cell expansion, early and late neurogenesis, gliogenesis, and aging [29,40–43]. A total of 70 ChIP-seq data sets, 14 DNase-seq data sets, 14 Hi-C data sets, 14 DNA methylation data sets and 14 RNA-seq data sets were generated (Table S2). For ChIP-seq, the patterns of H3K4me3, H3K27me3, H3K27ac, Pol2 and CTCF were profiled. The qualities of these data sets were assessed. The biological replicates displayed high correlation coefficients (Pearson's coefficients, 0.76–0.99) (Fig. S1a and b). The active epigenetic signals were clustered together, negatively correlated with the repressive signal (Fig. S1a and c). The genomic distribution of these epigenetic signals is consistent with previous reports (Fig. S1d–i) [31,37,44–50]. For example, the peaks of H3K4me3, H3K27ac and H3K27me3 are enriched in the promoter regions (TSS ± 2 kb). Moreover, the genes with active epigenetic signals show significantly higher expression levels than those without (Fig. S1j–l). Collectively, the epigenome and transcriptome data of rhesus PFC are robust and reliable for exploring the epigenetic regulatory mechanisms during PFC development in primates.

### Stage-specific epigenetic signals are associated with PFC development

Next, we assessed the dynamics of epigenetic signals for all genes during PFC development. Based on the active epigenetic signals of genes (see Methods), the rhesus PFC samples are classified into four major clusters, representing four distinct developmental and aging states of the nervous system, including neurogenesis, neuron migration and differentiation, glial proliferation and neural network establishment, and aging (Fig. S2a). Our data show that many genes harbor stage-specific epigenetic signals including chromatin higher-order structure, DHS, H3K4me3 and Pol2 (Fig. 1b and Fig. S2b). Notably, the signals of these stage-specific epigenetic modifications are highly consistent with each other. Moreover, the majority of genes with stage-specific epigenetic signals are stage-specifically expressed (Fig. 1b and Table S3). In total, we identified 11 703 genes (including 9338 coding genes and 2365 non-coding genes) with stage-specific epigenetic signals, which were referred to as stage-specifically regulated genes (SRGs). Importantly, the functions of these SRGs are consistent with the developmental events of the PFC at the corresponding stages (Fig. 1b and Fig. S2c). For example, SRGs at the E50 stage are enriched in cell cycle, signaling pathways of Hedgehog, Notch and WNT, and neuronal stem cell maintenance. SRGs at the E90 stage are enriched in neuron differentiation and cell migration. SRGs at the E120 stage are enriched in neuron projection development, cell migration and gliogenesis. SRGs at the P0 stage are enriched in myelination, neuron projection and regulation of axonogenesis. SRGs at the P4M stage are enriched in synapse organization and neurotransmitter secreting. SRGs at the PY4.5 stage are enriched in metal ion binding, neurotransmitter-gated ion channel clustering and neurotransmitter reuptake. SRGs at the PY20 stage are enriched in complement C3 and C4 levels, congenital disorders of metabolism, and the stress-induced intrinsic apoptotic signaling pathway. We also noticed many genes maintaining stable epigenetic signals across different developmental stages (Fig. S2d and e). The results from the Gene Ontology (GO) analysis indicate that these genes are significantly enriched in fundamental biological processes, such as the metabolic process and signal transduction (Fig. S2e). This is in contrast to the genes with stage-specific epigenetic signals in PFC, which tend to play specific functions in neural development.

### The dynamics of higher-order structures are associated with PFC development

The higher-order structure of chromatin partitions the genome into distinct functional domains, and mediates direct interactions between enhancers or repressors and promoters, affecting gene expression. To investigate how the dynamics of chromatin structure are involved in PFC development, we characterized the A/B compartments at mega base scale in rhesus PFC. Less than 16.7% of compartment regions undergo A/B compartment switches between adjacent stages (Fig. 1c and Fig. S3a–c). Our data indicate that 3480 genes in the switched compartments (from A to B or B to A) show dynamic gene expression (Fig. 1c and Table S4). Genes located in the compartments that change from A to B tend to be repressed, while those located in the compartments that change from B to A tend to be transcriptionally active (Fig. 1c). For example, GLI3, a well-known mediator of SHH signaling, can inhibit the differentiation of radial glial cells (RGCs) to glial cells, and maintain the bRG population [19,20]. Our data show that *GLI3* is located in an A compartment before the E90 stage, but in a B compartment afterwards (Fig. S3d). NEUROD6 is important for neuronal differentiation and axonal navigation, specifically expressed in the deep layers of the neocortex [51,52]. Our data show that *NEUROD6* is located in an A compartment at the E50 and E90 stages, but switches to a B compartment at the E120 stage (Fig. S3e). The results suggest that compartment switching is associated with neural cell differentiation in the PFC.

We further analyzed the dynamics of TADs during PFC development. The number of TADs ranges from 2720 to 3591 during rhesus PFC development, with ∼12% of TAD boundaries being dynamic (Fig. S4a and b). This finding is consistent with previous reports that TADs are conserved across various cell types and developmental stages [53,54]. We used TADCompare to identify the types of TAD alterations at each developmental stage by comparing the TAD signals between adjacent developmental stages [55]. The numbers of TADs undergoing TAD merge or split during development surpass those undergoing TAD shift, complex rearrangement, or strength change (Fig. 1d). Given that the dynamics of TADs coincide with changes in TAD boundaries, to investigate the relationship between TAD dynamics and PFC development, we identified dynamic TAD boundary-associated genes (dTADBAGs), located between 80 kb upstream and 80 kb downstream of dynamic TAD boundaries. Of these genes, 14.3%–46.7% show stage-specific or dynamic expression (Fig. S4c and d). Notably, genes with stage-specific or dynamic expression are predominantly found in dynamic TAD boundaries rather than stable TAD boundaries from the E90 stage onwards (Fig. S4e). Furthermore, those dTADBAGs with stage-specific expression are enriched in biological functions associated with PFC development (Table S5). These results suggest that alterations in TADs or TAD boundaries play important roles in regulating PFC development. For example, ARHGAP11A, which contains a Rho-GAP domain, is expressed in the basal end-feet of RGCs and controls their morphology and neuronal positioning [56]. In rhesus PFCs, *ARHGAP11A* is highly expressed at the E50 stage and decreases at the E90 stage, but not expressed at the E120 stage (Fig. S4f). Hi-C data reveal that *ARHGAP11A* is located within a TAD boundary with CTCF signals at both the E50 and E90 stages (Fig. 1e). However, at the E120 stage, the TAD boundary is relocated to a region 30 kb upstream of its original position. Accompanied by TAD boundary shift the, CTCF signal is shifted from site 1 to site 2 at the E120 stage (Fig. 1e). This shift leads to frequent interaction between the newly formed TAD boundary and *ARHGAP11A* promoter (Fig. 1e and Fig. S4f). Meanwhile, the expression of *ARHGAP11A* is repressed at the E120 stage. This implies the existence of a repressor binding site within the newly formed TAD boundary, which could potentially suppress the expression of *ARHGAP11A* through transcriptional repressors. We further investigated the conservation of this newly formed TAD boundary at the E120 stage. The results indicate that the orthologous region of the newly formed TAD boundary is conserved in the human genome but not the mouse genome, suggesting that this TAD boundary is primate-specific (Fig. S4g).

Previous studies have revealed that transcriptionally active human endogenous retrovirus subfamily H (HERV-H) is involved in the formation of new TAD boundaries [57]. To investigate the contribution of transposons to the establishment of new TAD boundaries during PFC development, we analyzed the enrichment of transposons at the newly established TAD boundaries at each developmental stage, and the expression levels of these transposons at the corresponding stage. Our results show that various types of transposons are enriched within the newly formed TAD boundaries, including L1, ERVL-MaLR, Alu, SVA and ERVL (Fig. S4h). In addition, L1, Alu and SVA are highly expressed in the PFCs at the E50 and E90 stages (Fig. S4i). This suggests that the active L1, Alu and SVA are associated with the establishment of new TAD boundaries. Furthermore, we examined the enrichment of TF binding motifs in the transposons located at the newly formed TAD boundaries. Interestingly, we observed that the binding motifs of ESR2 and NEUROND2 were enriched in L1 transposons at the E90 stage, whereas the binding motifs of TP53 and TFAP2C were enriched in L1 at the PY20 stage (Fig. S4j). Consistently, these TFs are expressed at the corresponding stages (Fig. S4j). ESR2, also known as NR3A2, is one of the estrogen receptors that is activated by the sex hormone estrogen. This implies that the sex hormone estrogen may influence TAD dynamics at the E90 stage. TP53 is a well-known factor involved in apoptosis. Notably, different TFs binding to the same types of transposons in newly formed TAD boundaries at different developmental stages were also observed for Alu (Fig. S4k). These results indicate that the binding of different TFs to these transposons and transposon activation at early embryonic and aging stages is associated with the formation of TAD boundaries.

We further investigated how epigenetic signals were changed in TADs during development and aging. For each epigenetic signal, we calculated the signal correlation between genomic bins within the same TAD and between genomic bins within different TADs (see  Supplementary Methods). Our results show that the intra-TAD correlation is higher than the inter-TAD correlation (Fig. S5a–e). Notably, both intra-TAD and inter-TAD correlations of Pol2, H3K4me3 and H3K27me3 significantly decrease after the P0 stage. This indicates that co-regulation of these signals in TAD is significantly reduced after the P0 stage. One possible reason is that the insulation function of the TAD boundary is weakened at this time point, which is supported by the decrease of CTCF signal from the P0 to P4M stages (Fig. 1f). Consistently, our results also indicate that the ratio of intra-TAD interaction to inter-TAD interaction decreases after birth (Fig. S1i). These findings suggest that the insulation strength of TAD boundaries is weakened after birth. Intriguingly, H3K27me3 signals across TAD boundaries can be frequently detected after birth, but rarely observed at embryonic stages (Fig. 1f). This phenomenon is associated with the decrease in insulation strength of TAD boundaries. To understand the regulatory roles of H3K27me3 signals across TAD boundaries, we analyzed the enrichment of genomic elements in the boundaries with H3K27me3 signals. Our results show that the transposons, including L1, Alu, ERVK and SVA, are enriched in this type of TAD boundary compared to random TAD boundaries (Fig. S5f). Furthermore, the H3K27me3 signal in the transposons located at TAD boundaries is high at the P0 and P4M stages compared to that at other developmental stages (Fig. S5g). We also observed that the expression levels of transposons are lower at the P0 and P4M stages compared to the E50, E90 and PY20 stages (Fig. S4i). This result suggests that H3K27me3 signals across TAD boundaries may repress the activities of transposons after birth.

### Epigenetic states of *cis*-regulatory elements are involved in the regulation of PFC development

Chromatin epigenetic states play important roles in regulating gene expression. To enhance the interpretation of these chromatin states, we defined a gene active signal (GAS) by integrating the signals of H3K4me3, H3K27ac, Pol2, H3K27me3 and DHS within promoter or gene body for each gene (see  Supplementary Methods). The GAS was calculated as the aggregate of normalized active signals (H3K4me3, H3K27ac, Pol2 and DHS) minus the normalized repressive H3K27me3 signal. 12 571 genes with stage-specifically high GASs were identified (Fig. 1g). To assess the reliability of this method, we examined the expression of these genes. As expected, these genes with stage-specifically high GASs show stage-specific expression (Fig. 1g). We further investigated the functions of the genes with stage-specifically high GASs through enrichment analysis using published marker genes of human neural cell types [58] (Table S6). Our data show that the genes with stage-specifically high GASs at the E50 stage (herein referred to as E50 specific GAS) are enriched in the marker genes of neural progenitor cells (NPCs), the genes with E90 and E120 specific GASs are enriched in the marker genes of excitatory neurons, the genes with E120 and P0 specific GASs are enriched in the marker genes of interneurons and oligodendrocyte precursor cells (OPCs), the genes with PM4 to PY20 specific GASs are enriched in the marker genes of astrocytes, and the genes with PY20 specific GASs are enriched in the marker genes of microglia (Fig. 1h). This suggests that the establishment of active states in promoters at specific stages is crucial for neuronal and glial cell differentiation in the brain during development.

To explore the roles of genes with active signals at various stages during PFC development, we conducted a comparative analysis between these genes and different gene sets. These gene sets consist of neuronal disease-associated TFs, neuronal disease-associated genes, all disease-associated genes, protein coding genes, and all genes (Table S7). The results show that many genes with active signals in PFCs are neuronal disease-associated genes or TFs (Table S8). We were curious about whether these genes with active signals played important roles at early or late developmental stages. Therefore, we performed the cumulation fraction analysis of the genes with active signals within different gene sets across developmental stages (see Supplementary Methods). Our data show that the neuronal disease-associated TF genes accumulate significantly faster than the other gene groups (Fig. 1i, 1.82-fold, *p* = 2 × 10^−7^ compared to the gene set of all genes). This suggests that the neuronal disease-associated TFs are likely to play important roles in early PFC development.

Previous works reported that the gene expression was dynamic during the development of PFC, with a notable high rate of change occurring at early stages. This rate then decreased postnatally, reaching a relatively stable state by the young adulthood stage [29,40]. Consistently, the dynamic rate of gene expression shows an ‘hourglass’ pattern, with the lowest dynamic rate observed around the birth stage (Fig. 1j). To determine whether the dynamics of epigenetic signals are associated with the changes in gene expression, we examined the dynamic of epigenetic signals by calculating the proportion of genomic regions with the epigenetic signals undergoing signal changes at subsequent developmental stages. Unexpectedly, contrary to the dynamic rate of gene expression, the changes in epigenetic signals are most pronounced around the birth stage from P0 to P4M stages and decrease throughout postnatal development and aging (Fig. 1j). This indicates that the dramatic changes of epigenetic signals around the birth stage do not significantly influence the expression of genes at the corresponding developmental stage (Fig. S6a). Notably, we noticed that many genes that established H3K4me3 signals in their promoters at the P0 stage were not expressed at this stage, but expressed at the P4M stage (Fig. S6b). This suggests that there is a priming mechanism of epigenetic signals in regulating gene expression at the birth stage. Further investigation revealed that the expression of these primed genes at the P4M stage was associated with an increase in chromatin interaction (Fig. S6c).

In addition to gene activation, the expression of genes can be repressed through repressive epigenetic modifications such as DNA methylation and H3K27me3. We further assessed gene repression during PFC development. Our findings reveal that for the promoters with stage-specific H3K27me3 signals during PFC development, a majority of these promoters (*n* = 8046) show a transition tendency from high H3K27me3 signals to DNA hypermethylation at subsequent postnatal stages (Fig. 1k and Table S9). This suggests that DNA methylation progressively replaces the H3K27me3 signals in promoters to repress the expression of some genes at postnatal stages.

Taken together, these results indicate that establishment of active epigenetic signals is associated with the activation of genes participating in PFC development. The dramatic dynamic of epigenetic states occurs around the birth stage.

### Regulatory modules of NPCs during neurogenesis

**Figure 2.**
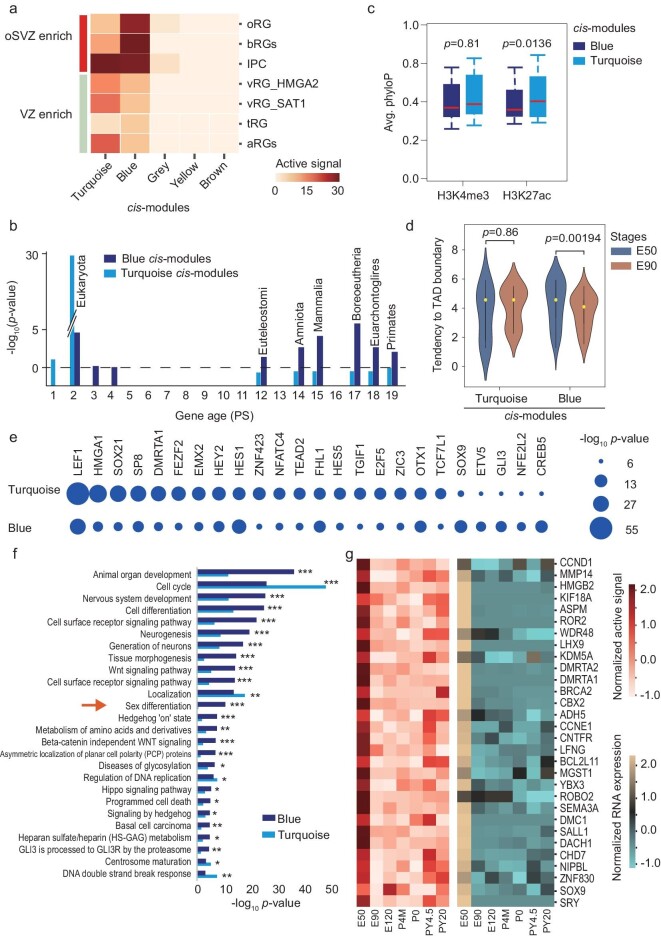
The *cis*-element modules associated with bRGs and aRGs during PFC development. (a) Heat map showing the enrichment of the genes associated with *cis*-element modules in marker genes of different types of neural progenitor cells (NPCs). The *cis*-element modules were classified by using WGCNA. The marker genes of rhesus NPCs were obtained from a previously published study [30]. oRG, outer radial glia (RG); bRG, basal RG; IPC, intermediate progenitor cell, vRG, ventricular radial glia; tRG, transient RG; aRG, apical RG; oSVZ, outer subventricular zone; VZ, ventricular zone. vRG, tRG and aRG are enriched in VZ. *cis*-module represents *cis*-element module. (b) Bar plots showing the enrichment of the genes associated with the blue and turquoise *cis*-modules in the genes with different evolutionary ages. PS, phylostrata. (c) Box plot comparing phyloP scores of *cis*-elements in the blue and turquoise modules. Wilcoxon rank sum test is used. (d) Violin plot showing the location tendency of the genes associated with the turquoise and blue *cis*-element modules to TAD boundaries at the E50 and E90 stages. Wilcoxon rank sum test is used. (e) Dot plot of transcription factor binding motif enrichment in the blue and turquoise *cis*-element modules. Size of dot represents enrichment score. These transcription factors were reported to be expressed in RGs. (f) Bar plot of GO enrichment of the genes associated with the blue and turquoise *cis*-element modules. (g) Heat map showing the normalized active epigenetic signals and RNA expression of the genes associated with the blue *cis*-module. These 28 genes are involved in sex differentiation. For a given gene, the active epigenetic signals in both promoter and *cis*-elements were used to calculate its active epigenetic signals. The blue *cis*-element module is a bRG-associated *cis*-module.

A significant characteristic of primate evolution is the substantial increase in brain size. This can be attributed to the proliferation of bRGs in the primate brain, which are generated from the differentiation of apical radial glia (aRG) cells and significantly augment the number of neuronal cells and cell types [13–18]. The cell cycle duration for aRGs is significantly shorter than that for bRGs [12]. Until now, the differences in epigenetic regulatory mechanisms between aRGs and bRGs is still largely unknown. To explore these epigenetic regulatory mechanisms, we identified 2639 *cis*-elements whose active score dynamics correlate strongly with the expression changes of associated genes during PFC development. These *cis-*elements, including promoters and enhancers, were further classified into five major *cis*-element modules using weighted gene co-expression network analysis (WGCNA). These *cis*-element modules consist of the blue module (*n* = 1070), turquoise module (*n* = 1361), brown module (*n* = 52), gray module (*n* = 156) and yellow module (*n* = 31) (Fig. S7a–c). To find out which *cis*-element modules were associated with bRG and aRG development, we analyzed the enrichment of *cis*-element module-associated genes in the genes involved in rhesus bRG [12,16,17,30] and aRG [12,18,30] development, respectively. Our results show that the genes associated with the blue module are enriched in those genes involved in bRG development but not aRG, while the genes associated with the turquoise module are enriched in those genes participating in aRG development but not bRG (Fig. 2a). Therefore, we referred the blue module to the bRG-associated *cis*-element module, and the turquoise module to the aRG-associated *cis*-element module. Furthermore, we examined the evolutionary ages of these genes associated with the *cis*-element module. The evolutionary ages of genes can be classified into different phylostrata (PS) by using a phylostratigraphic approach [59]. PS values represent different levels of the taxonomic hierarchy in the tree of eukaryotic life when the genes emerge. The smaller the PS value, the older the gene. Our results indicate that the genes associated with the bRG-associated *cis*-element module are enriched in the evolutionarily young genes, whereas those associated with the aRG-associated *cis*-element module are enriched in evolutionarily old genes (Fig. 2b and Table S10). In particular, the enrichment of bRG *cis*-module-associated genes in PS15 (mammalia) and PS17 (boreoeutheria) is particularly high, which coincides with the expansion of brain size in mammalia. We further characterized and compared the *cis*-element modules associated with bRGs and aRGs. The placental mammals phyloP scores were used to evaluate the conservation of the *cis*-element modules, including promoters and enhancers. Our results show that the phyloP scores of promoters in the bRG-associated *cis*-element module are comparable to those in the aRG-associated *cis*-element module, while the phyloP scores of enhancers in the bRG-associated *cis*-element module are lower than those in the aRG-associated *cis*-element module (Fig. 2c). This suggests that the bRG-associated enhancers are less conserved than the aRG-associated enhancers, implying a reduced evolutionary constraint on the bRG-associated enhancers. Furthermore, genes associated with the bRG- rather than aRG-associated *cis*-element module tend to be located at TAD boundaries at the E50 stage (Fig. 2d). To determine which TFs contribute to the development of bRGs and aRGs, we analyzed the enrichment of TF bind sites in promoters (marked by H3K4me3) and enhancers (marked by H3K27ac) within the *cis*-element module. Only the TFs reported to be expressed in rhesus RGs were analyzed [12,18,30]. The binding motifs of HES1, LEF1 and OTX1 are enriched in these two *cis*-element modules. (Fig. 2e). Additionally, the binding motifs of CREB5, GLI3, ETV5 and SOX9 are more enriched in the bRG-associated *cis*-element module (blue module) than aRG-associated *cis*-element module (turquoise), while the binding motifs of ZNF423, TEAD2, TGIF1 and TCF7L1 are more enriched in the aRG-associated *cis*-element module than bRG-associated *cis*-element module (Fig. 2e). Among them, GL3 is a well-known transcription factor involved in bRG development.

We also investigated the biological functions of genes associated with bRG- and aRG-associated *cis*-element modules using GO enrichment analysis. The results show that genes associated with the bRG-associated *cis*-element module are enriched in nervous system development, Wnt and Hedgehog signaling pathways, and notably asymmetric localization of planar cell polarity (PCP) proteins, which is in line with the fact that bRGs undergo asymmetric cell divisions. In contrast, genes associated with the aRG-associated *cis*-element module are enriched in DNA double-strand-break response, centrosome maturation, regulation of DNA replication and cell cycle. This is consistent with the fact that aRGs undergo rapid cell division and are susceptible to DNA damage. It is interesting that genes associated with the bRG-associated *cis-*element module are enriched in sex differentiation (Fig. 2f and g). In particular, the sex-determining region Y protein (*SRY*) and *SOX9*, involved in male sex development, both exhibit active epigenetic signals at the E50 and P0 stages (Fig. S7d and e). Notably, genes associated with the bRG- but not aRG-associated *cis-*element module include a primate-specific TF ZNF830 (Fig. 2g). These results indicate that a *cis*-regulatory element network is associated with both bRG development and sex determination.

Primate NPCs are believed to accumulate genetic heterogeneity through retrotransposon activity [60]. To identify the retrotransposons involved in this process, we examined the RNA expression of retrotransposons from E50 to PY20 stages. The results show that a variety of retrotransposons, especially Alu, ERVK, SVA and L1, show high expression levels at the E50 stage when neocortical expansion occurs (Fig. S4i). We further analyzed the epigenetic active scores of these retrotransposons at the E50 stage. These results indicate that active epigenetic signals are enriched in L1, SVA, Alu, ERVL-MaLR, etc. (Fig. S7f). For example, the active signal of an L1 is highest at the E50 stage and gradually decreases thereafter (Fig. S7g).

Taken together, aRG and bRG are regulated by distinct *cis-*element modules. These differences correlate with the varied cellular features of these two types of NPCs.

### Bivalent genes pre-configured cell differentiation and layer specification

**Figure 3.**
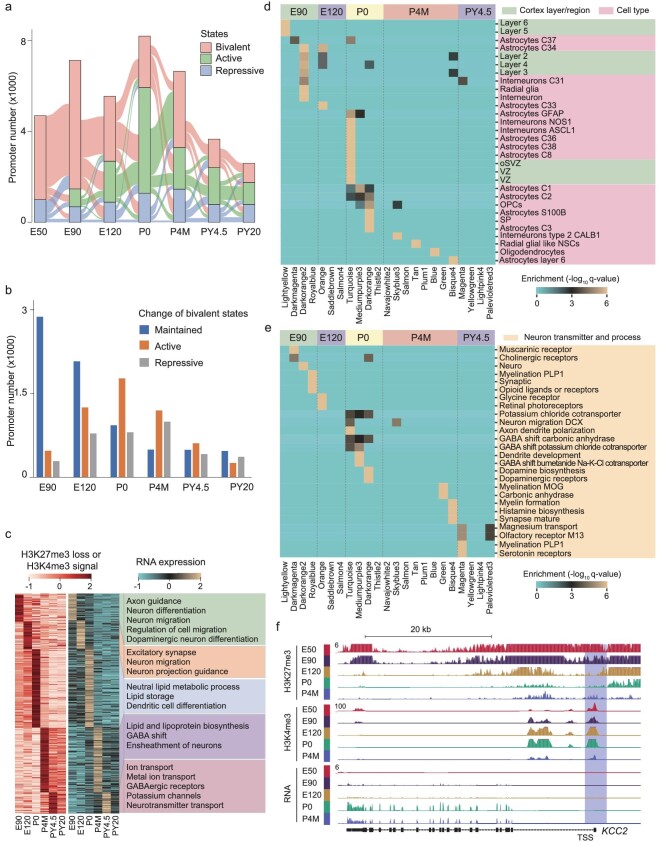
The dynamic of bivalent histone modifications is associated with PFC development. (a) Alluvial plots showing dynamics of epigenetic states of promoters during PFC development. The heights of bars represent the numbers of promoters or genes. Bivalent promoters harbor both H3K4me3 and H3K27me3 signals. Active promoters harbor only H3K4me3 signals. Repressive promoters harbor only H3K27me3 signals. (b) Bar plot showing the numbers of genes that maintain bivalent promoters, or change from bivalent promoters to active or repressive promoters at different stages compared to prior developmental stages. (c) Heat map showing the H3K4me3 and RNA expression signals of genes whose promoters change from bivalent states to active states at each stage. The GO terms of these genes are shown on the right. (d) Heat map showing the enrichment of WGCNA gene modules of BTA genes gaining active signals at different development stages in the gene sets associated with neuronal differentiation and layer specification. BTAs represent the genes whose promoters change from bivalent states to activate states. (e) Heat map showing the enrichment of WGCNA gene modules of BTA genes gaining active signals at different development stages in the gene sets associated with neuron transmitter and related processes. (f) Genome browser view of H3K4me3, H3K27me3 and RNA expression signals at *KCC2* gene locus. The promoter of *KCC2* is denoted by a shadow.

Bivalent promoters, characterized by both H3K4me3 and H3K27me3 signals, are reported to play important roles in cell fate determination [61]. Many developmental genes are primed with bivalent signals within their promoters at initial stages, subsequently undergoing rapid activation during development. However, the regulatory functions of these bivalent promoters during PFC development are still largely unknown. To answer this question, we investigated the dynamics of bivalent promoters in rhesus PFCs. The numbers of these bivalent promoters are changed dramatically during PFC development. At prenatal stages, particularly E50 and E90 stages, ∼78.5% of genes are marked by bivalent signals in their promoters (Fig. 3a). During PFC development, although many bivalent promoters are maintained into subsequent stages, the proportions of these maintained bivalent promoters decrease (Fig. 3b). Concomitantly, a substantial portion of bivalent promoters become active promoters with only H3K4me3, or repressive promoters with only H3K27me3. The majority of bivalent promoters are changed to active promoters at the P0 stage (Fig. 3a and b). Consistently, the expression levels of genes whose promoter transitions from bivalent at previous stages to active states (referred to as BTA genes) significantly increase at the corresponding stages (Fig. 3c). To explore the functions of these BTA genes, we performed GO enrichment analysis for these genes. BTA genes at the E90 and E120 stages are enriched in neuron migration (Fig. 3c). It is consistent with previous reports that the migration events occur at these stages [29,62]. We further explore the functions of these BTA genes in neurodevelopment. These BTA genes were classified into different modules using WGCNA. Subsequently, the enrichment of the gene module in the gene sets reported to be involved in neuronal differentiation and functions was analyzed. Some BTA genes at the E90 and E120 stages participate in the specification of deep layer neurons (*FEZF2, BCLL11B, FOXP2*), or in the dendritic branching and synapse formation in the upper layers (*CUX1, CUX2*) (Fig. 3d). Notably, some BTA genes at the E120 stage (*CTXN3, MEF2C, CTGF, SATB2, PCDH8, OPRK1*) are corticothalamic extra-telencephalic markers, contributing to the expansion of layers II–VI. These results suggest that bivalent signals can prime the expression of the genes involved in PFC layer, corticocortical and corticothalamic specification. BTA genes at the E90 and E120 stages are also enriched in excitatory neurogenesis. In addition, BTA genes at the E90, E120, P0 and especially P4M stages are enriched in interneuron and dendrite development, which contribute to the memory and learning function for synapses at these stages. BTA genes at the P0 and P4M stages are enriched in the development of astrocytes, OPCs and microglia. These results suggest that many genes participating in brain cell-fate determination are also marked by bivalent promoters at earlier stages. Intriguingly, SOX5, FOXP2 and CTGF are critical for the development of layer-dependent corticofugal connectivity (Layer VIb), and belong to BTA genes at the E90 stage. This implies that the pre-configuration of corticofugal wiring begins as early as the E90 stage, corresponding to the major neuron differentiation stage (Fig. 3d).

The GABA shift is a crucial neurodevelopmental event in which GABA is converted from an excitatory to an inhibitory neurotransmitter. Our results indicate that the GABA-shift-associated genes are enriched in the BTA genes at the P0 stage (Fig. 3e). KCC2 is a K^+^-Cl^−^ cotransporter controlling the GABA shift [63]. Our data show that the promoter of KCC2 is bivalent at the E50, E90 and E120 stages, but changed to an active state with only H3K4me3 but not H3K27me3 signal at the P0 stage (Fig. 3f). In addition, KCC2 is highly expressed at the P0 and P4M stages, which are reported to be key stages for GABA shift (Fig. 3f). Thus, genes involved in the GABA shift of PFC are primed by bivalent promoters at early stages, which are subsequently activated from the date of birth onwards.

Taken together, numerous promoters of significant genes involved in neurodevelopment are primed with bivalent signals in their promoters at earlier stages before their activation through a decrease of H3K27me3 signals and an increase or maintenance of H3K4me3 signals at specific stages during PFC development. The transition from bivalent state to active state can fit the requirements of neural development, including layer specification, neural cell differentiation, dopaminergic neuron maturation and GABA shift.

### The formation of super enhancers is associated with neural network wiring

**Figure 4.**
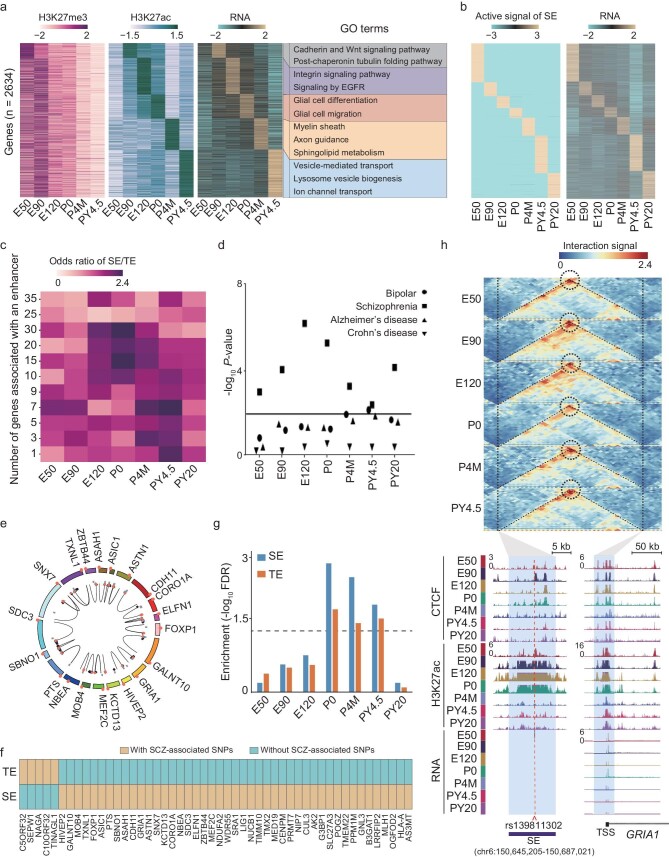
Super enhancers (SEs) in PFCs during development. (a) Heat map showing H3K27me3 and H3K27ac signals of stage-specific putative enhancers and gene expression levels of the genes associated with the putative enhancer in rhesus PFC during development. The enriched GO terms for the genes associated with putative enhancers are shown on the right. (b) Heat map showing H3K27ac signals of SEs and RNA expression levels of the genes associated with SEs during PFC development. The H3K27ac signal of an SE was calculated by summing the values of -log (ppois *pvalue*) within the SE. (c) Heat map showing the odds ratio of SE number to typical enhancer number in each enhancer group, classified according to the numbers of their potential target genes at different stages. (d) Dot plot showing enrichment of genes associated with SEs in the genes associated with various types of neuronal diseases (bipolar, schizophrenia, Alzheimer) and a non-neuronal disease (Crohn's disease). The horizontal line represents *P* = 0.01. (e) Circos plot showing the previously unreported SCZ-associated genes whose SEs harbor SCZ-associated SNPs. (f) Heat map showing the SCZ-associated genes associated with SEs and typical enhancers. (g) Bar plot comparing the enrichment of genes associated with SEs and typical enhancers in the genes associated with intelligence. The dashed line represents False Discovery Rate (FDR) = 0.05. (h) Genome browser view of CTCF, H3K27ac signals and RNA expression signal at *GRIA1* locus and its SE locus. *GRIA1* is an SCZ-associated gene. The regions of GRIA1 promoter and SE are labeled in shadows. *rs139811302* is an SCZ-associated SNP.

During PFC development, synapse pruning and neural network establishment mainly occur in the first several weeks after birth. These processes are regulated in a stage-specific manner. Previous studies reveal that enhancers play important roles in regulating stage-specific gene expression and cell fate determination. However, the role of enhancers in the establishment of the neural network is still unclear. To identify enhancers in rhesus PFCs throughout development, we integrated H3K27ac ChIP-seq, DNase-seq, Hi-C and CTCF ChIP-seq data. If the distal *cis*-elements were located within 3 megabases (Mb) upstream and downstream of transcription start sites, but not in promoters, exhibited signals of H3K27ac, CTCF, and DHS, and harbored chromatin interactions with promoters, they were defined as putative enhancers. In total, 42 883 putative enhancers exhibiting loop interactions with promoters were identified. Of them, between 14 517 to 23 511 putative enhancers are identified at each developmental stage. We firstly identified stage-specific and stage-common enhancers. There are 108 110 stage-specific enhancers and 4527 stage-common enhancers across the developmental stages investigated in this study (Fig. S8a and b). To understand how these stage-specific putative enhancers regulate stage-specific gene expression during PFC development, we checked the expression of the genes associated with the stage-specific putative enhancers. Our results show that numerous putative enhancers (*n* = 2634) are marked by the H3K27me3 signal during prenatal stages, while these enhancers are marked by H3K27ac but not H3K27me3 signal during postnatal stages, and are associated with stage-specific gene expression (Fig. 4a). This suggests that the activities of many enhancers regulating stage-specific expression are repressed by H3K27me3 signals at earlier stages. The genes associated with the E90-specific putative enhancer are enriched in the cadherin and WNT signaling pathways, and the post-chaperonin tubulin folding pathway. The genes associated with the E120-specific putative enhancers are enriched in the integrin signaling pathway and the EGFR signaling pathway. The genes associated with the P0-specific putative enhancers are enriched in glial cell migration and differentiation. The genes associated with the P4M-specific putative enhancers are enriched in myelin sheath, axon guidance and sphingolipid metabolism. The genes associated with the PY4.5-specific putative enhancers are enriched in vesicle-mediated transport, lysosome vesicle biogenesis and ion channel transport (Fig. 4a). Furthermore, the genes associated with the stage-common putative enhancers are enriched in metabolism, membrane trafficking, cell cycle, etc. (Fig. S8b). Interestingly, we observe that ∼50% of the putative enhancers show loop interaction with a single promoter. However, 25% of the putative enhancers exhibit interaction with more than two promoters, implying that these enhancers may serve as regulatory hubs (Fig. S8c). We further explored the TF binding motif enrichment in the putative enhancers. The results show that the binding motifs of PAX6 and EOMES are enriched in the E50-stage specific *cis*-elements; the binding motifs of CUX2, NEUROD1 and MEF2C are enriched in the stage-specific *ci*s-elements from E90 to P0; and the binding motifs of FLI1 and FOXA2 are enriched in the stage-specific *cis*-elements at the P4M and PY4.5 (Fig. S8d). These TFs are highly expressed at the corresponding stages (Fig. S8d). The binding motifs enriched in the stage-common enhancers include CTCF, ZIC3 and OLIG2 (Fig. S8e).

Previous work has revealed that the majority of super enhancers (SEs) exert cell-type specific functions [64]. To understand the functions of SEs in PFC development, we classified the putative enhancers into typical enhancers (TEs, length <8 kb) and SEs (length >=8 kb) based on their sizes [64]. The signals of SEs and the expression of SE-associated genes show stage-specific patterns during PFC development (Fig. 4b and Table S11). We notice that the number of SEs in all enhancers is the highest at the P0 stage when the neural network is establishing (Fig. S8f). Furthermore, at the P0 stage, SEs rather than TEs tend to target or be associated with 15–30 genes (Fig. 4c).

To answer the question of whether the abnormalities of SEs lead to defects of neural network wiring, we focused on schizophrenia (SCZ), one of the most prevalent developmental diseases of the nervous system. At the onset of SCZ symptoms [65] are thought to be disorders caused by developmental dysregulation of neural network wiring through reduced synaptic connectivity [66,67]. We first evaluated the enrichment of SCZ-associated single nucleotide polymorphisms (SNPs) in SEs. To minimize false positive detection, we mainly focused on SEs with high correlation (r >= 0.8) between their epigenetic signals and the expression levels of their associated genes. Our results show that SCZ-associated SNPs are enriched in the SEs at all stages, especially the E120 and P0 stages (Fig. 4d). In addition, SNPs associated with bipolar disorder are also significantly enriched in SEs at the PY4.5 stage. In contrast, Alzheimer’s disease (AD) and Crohn's disease (a non-psychiatric disease) associated SNPs are not enriched in SEs at any stage (Fig. 4d). In total, 391 distal SEs harbor SCZ-associated SNPs. These SEs are associated with 63 genes. It is noteworthy that 20 out of the 63 genes have not been reported to be involved in SCZ development (Fig. 4e). We are curious about whether abnormal SEs contribute more significantly to SCZ than abnormal TEs. We compared the distribution of SCZ-associated SNPs in SEs and TEs. Forty-five SNPs are located in SEs, while only five SNPs are located in TEs. The enrichment of SCZ-associated SNPs is higher in SEs than TEs (odds ratio = 2.89, *P*-value = 0.0243) (Fig. 4f). The functions of these SE-associated genes include neurotransmitter receptors, extracellular matrix proteins or adherence factors, myelin, and postsynaptic membrane proteins that are footstones for neural firing and wiring [68] (Fig. 4f). In addition, we obtained the genes associated with intelligence through a linkage disequilibrium score regression analysis (*P-*value < 0.05) utilizing published GWAS data for the intelligence trait. Our results show that the SE-associated genes are more enriched in intelligence than TE-associated genes at the P0, P4M and PY4.5 stages, all of which are critical for neural network establishment (Fig. 4g, Fig. 8g). For example, the *GRIA1* gene encodes glutamate ionotropic receptor AMPA type subunit 1, which is associated with the intelligence trait [69]. It harbors an SE from the E90 to P0 stages, and is highly expressed at these stages (Fig. 4h). The interaction between the SE and *GRIA1* promoter is supported by Hi-C interaction and CTCF signals. Notably, an intelligence-associated SNP (*rs139811302*) is located in the SE, supporting the important role of the SE in neuronal function.

Taken together, SEs around birth day stage (P0) are associated with neural firing and wiring during PFC development. Although both SEs and bivalent promoters transitioning are linked to the development of PFC, it is interesting that only a minor fraction of the SE-associated genes overlap with those genes regulated by bivalent promoters (Fig. 8h). But the overlap is significant at the E90, E120, P4M and PY20 stages. It implies that different types of *cis*-regulatory elements are coordinately employed to regulate the expression of genes involved in PFC development.

### A CRE regulates the expression of *DBN1* and neural development

**Figure 5.**
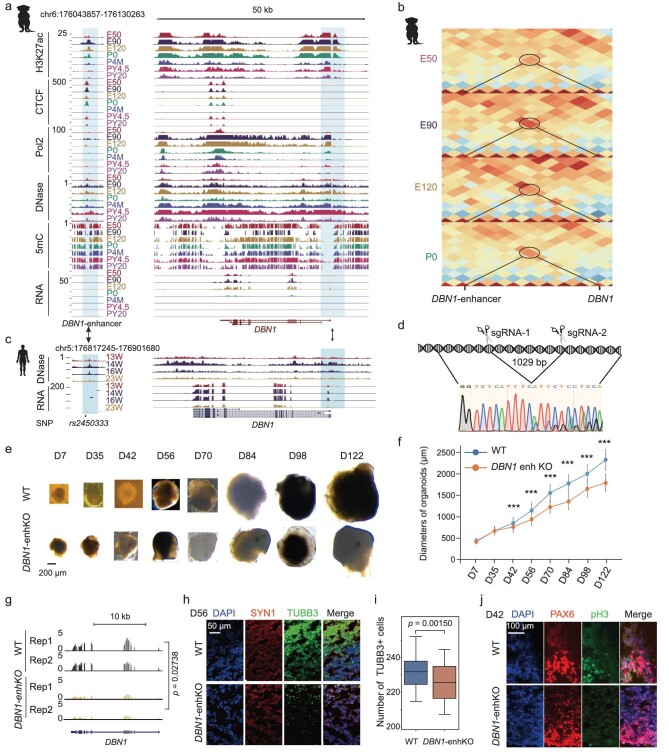
An enhancer of *DBN1* is critical for NPC proliferation and neuronal differentiation in human cortical organoids. (a) Genome browser view of epigenetic signals and RNA expression patten of *DBN1* and its putative enhancer during rhesus PFC development. The promoter and putative enhancer regions are denoted by shadows. (b) Heat map showing the loop interaction signal between the *DBN1* promoter and its enhancer during rhesus PFC development. (c) Genome browser view of DNase-seq and RNA-seq signals of *DBN1* in human PFC (13W, 14W, 16W and 23W). *rs2450333* is an SCZ-associated SNP. W, gestational weeks. (d) Carton showing the target sites of *DBN1* enhancer guide RNAs (gRNAs) and sanger sequence of genomic DNA of *DBN1* enhancer knockout (KO) H9 cells at *DBN1* enhancer locus. (e) Bright field images of cortical organoids originated from WT and *DBN1* enhancer KO H9 cells. Scale bar, 200 μm. Three organoids were used for measurement at each time point for each group. (f) Plot showing the sizes of cortical organoids originated from WT and *DBN1* enhancer KO H9 cells shown in (e). Three organoids were used for measurement at each time point for each group. Two-sided *t* test is used. Error bars represent mean$\ \pm $ s.e.m. *** represents *p* < 0.001. (g) Genome browser view of RNA-seq signal of *DBN1* genes in WT and *DBN1* enhancer KO cortical organoids at Day 56. (h) Immunostaining of SYN1, TUBB3 and DAPI in WT and *DBN1* enhancer KO cortical organoids at Day 56. Scale bar, 50 μm. Representative images are shown from *n* = 3 independent replicates. (i) Box plot comparing the numbers of TUBB3 + cells between WT (*n* = 3) and *DBN1* enhancer KO cortical organoids (*n* = 3). Two-sided *t*-test is used. (j) Immunostaining of PAX6, pH3 and DAPI in WT and *DBN1* enhancer KO cortical organoids at Day 42. Scale bar, 100 μm. Representative images are shown from *n* = 3 independent replicates.

To explore the regulatory functions of CREs in PFC development and disease, we focused on CREs whose associated orthologous genes in humans were linked to human neuronal diseases or traits, as evidenced by published GWAS data [70–72]. These orthologous genes include SCZ-associated genes, as well as intelligence- and educational-attainment-associated genes. The interaction between CREs and these associated orthologous genes was confirmed by our Hi-C loop results. We further compared the genomic sequences of CREs between rhesus monkeys and humans [73]. Only CREs with conserved sequence and active epigenetic signals in the PFC were selected for further investigation. Among them, we focused on a CRE of *Drebrin 1* (*DBN1*). DBN1 plays a key role in regulating the process of neuronal growth and differentiation [74]. It has been reported that mutations in DBN1 are associated with SCZ [75]. The CRE is located at a locus ∼320 kb upstream of *DBN1* and shows strong interaction with *DBN1* promoter at the E90 and E120 stages. Consistently, *DBN1* is highly expressed before the E120 stage (Fig. 5a and b). Consistently, DBN1 is expressed at gestation week (GW) 13, GW14, GW16 and GW23 stages in the human PFC (Fig. 5c).

Based on the conservation of the CRE-*DBN1* pair between rhesus and human, we investigated the function of the CRE in human cortical organoids, which can recapitulate human cortex development [73,76]. The CRE was knocked out in human H9 embryonic stem cells using the CRISPR/Cas9 system (Fig. 5d and Fig. S9). Subsequently, we assessed the cortical organoid derived from *DBN1* CRE KO H9 cells. The sizes of *DBN1* CRE knockout (KO) organoids were significantly smaller than those of control organoids at Day 42 and beyond (Fig. 5e and f). This indicates that this CRE plays an important role in the proliferation of cortical organoids. To verify the regulatory relationship between this CRE and *DBN1*, we checked the expression of *DBN1* in *DBN1* CRE KO cortical organoids at Day 56. As expected, the expression level of *DBN1* significantly decreased in the CRE KO organoid compared to the control group (Fig. 5g, t-test *P* = 0.02738). To further explore the functions of this CRE in neuronal differentiation, we evaluated the signals of synapsin1 (SYN1, a pre-synaptic marker) and TUBB3 (a neuronal cell marker) in cortical organoids by performing immunofluorescence staining at Day 56. Our results show that the number of synaptic connections between neurons in *DBN1* CRE KO organoids is less than that in control organoids (Fig. 5h and i, t-test, *P* = 0.00150). Notably, a human SCZ-associated SNP is detected in this CRE (Fig. 5c). To determine whether there is any defect in the proliferation of neural cells in the *DBN1* CRE KO organoids, we performed pH3 and PAX6 double immunostaining to assess the proliferation state of NPCs in the organoid at Day 42. The results show that there are obviously less pH3 + NPCs in DBN1 CRE KO organoids than in WT control organoids (Fig. 5j). This indicates that the proliferation of NPCs is defective in *DNB1* CRE KO organoids, which is responsible for the small size of the KO organoid. Taken together, our data suggest that the regulatory function of CRE in *DBN1* expression is crucial for the proliferation of NPCs during development.

### Changes in chromatin structure during brain aging

**Figure 6.**
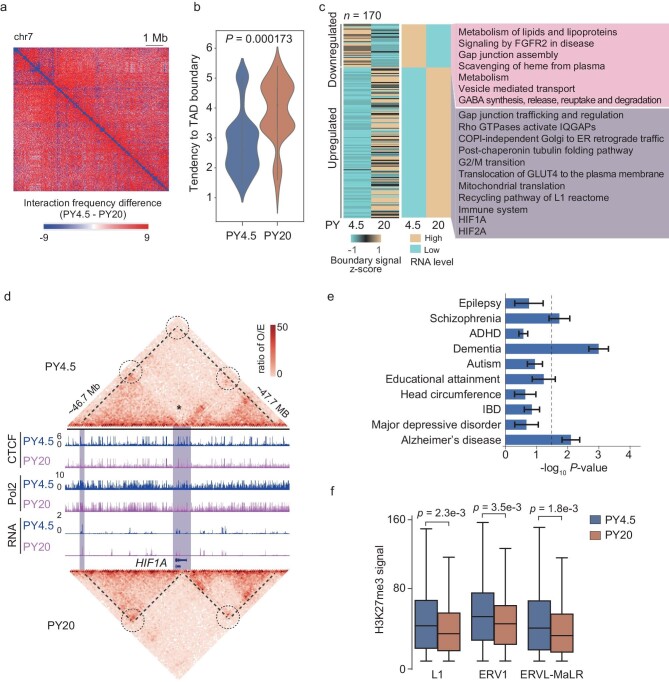
Short-range chromatin interaction increases and long-range chromatin interaction decreases during PFC aging. (a) Heat map showing the differences of chromatin interaction frequencies in rhesus PFC on chromosome 7 between the PY4.5 and PY20 stages. The differences in chromatin interaction frequency was calculated as the interaction frequency between two genomic regions at the PY4.5 stage minus that at the PY20 stage. (b) Violin plot comparing the tendency of gene location to the TAD boundaries for the genes differentially expressed at the PY20 stage compared to the PY4.5 stage in the split TADs. Wilcoxon rank sum test is used. *** represents *p* < 0.001. (c) Heat map showing the expression levels of genes located in the newly formed TADs and with differential expression levels between the PY4.5 and PY20 stages. The GO terms of these genes are shown on the right. (d) Genome browser view of chromatin interaction, CTCF, Pol2 and RNA expression signals across *HIF1A* locus. The regions of TADs are labeled by dashed lines. The interactions between TAD boundaries, which show changes from the PY4.5 stage to the PY20 stage, are shown in dashed circles. The asterisk indicates the location of the newly formed TAD boundary at PY20 stage. (e) Enrichment of genes losing interaction with distal enhancers at the PY20 stage in the genes associated with distinct types of neurodegeneration diseases. (f) Box plots comparing the H3K27me3 signals in different classes of retrotransposons between PY4.5 and PY20. Wilcoxon rank sum test is used.

The aging process of the brain is marked by changes in stereotypical structures and neurophysiological functions, as well as a varying degree of cognitive decline [77]. To explore the underlying regulatory mechanisms of brain aging, we compared epigenomes of PFCs between the PY4.5 and PY20 stage, representing adult and old stage, respectively. We compared the A/B compartments and TADs in PFCs between these two stages. From PY4.5 to PY20 stage, the number of compartments exhibiting A to B transition is more than those exhibiting B to A transition (Fig. 1c). The number of TADs increases from the PY4.5 to PY20 stage (Fig. S4a), meanwhile the median size of TADs decreases from 730 kb at the PY4.5 stage to 560 kb at the PY20 stage (Fig. S10a, t-test, *P* = 1.31 × 10^–3^). The proportion of promoter–enhancer interaction also decreases (Fig. S10b). Consistently, many TADs are split into smaller TADs at the PY20 stage (Fig. 1d, t-test, *P* = 2.2 × 10^–5^). Furthermore, the relative frequency of long-range interaction (>730 kb) decreases, while that of short-range interaction (<730 K) increases from the PY4.5 stage to PY20 stage (Fig. 6a and Fig. S10c and d). The differentially expressed genes at the PY20 stage compared to the PY4.5 stage tend to be closer to TAD boundaries (Fig. 6b). These results indicate that short-range interactions increase during brain aging. To confirm this observation, we assessed the alteration of contact probabilities and Hi-C count enrichment in different ranges of genomic regions during aging. As expected, the old brain displays an increase in contact probabilities at ≤1 Mb and a decrease in contact probabilities at >1 Mb distances compared with the young brain (Fig. S10d). A similar result is obtained by calculating Hi-C count enrichment in different ranges of genomic regions (Fig. S10c). To investigate whether the increase of short-range interaction and the decease of long-range interaction during brain aging is conserved in humans, we compared chromatin interaction in human PFCs between young (average age = 29) and old groups (average age = 90), utilizing published Hi-C data [78]. The results indicate that an increase of short-range interaction is also observed in aged human PFCs (Fig. 10e and f), suggesting that this phenomenon exhibits evolutionary conservation. The increase of short-range interaction during brain aging results in the split of TADs and the formation of new TAD boundaries.

To explore the influence of increased short-range interactions during brain aging, we analyzed the expression and functions of the genes within the newly formed TAD boundaries, which were induced by the increase of short-range interactions. Of these genes, 38 are downregulated and 132 genes are upregulated at the PY20 stage compared to the PY4.5 stage (Table S12). The downregulated genes are enriched in metabolism of lipids and lipoproteins, metabolism, vesicle-mediated transport, GABA synthesis, release, reuptake and degradation (Fig. 6c). In contrast, the upregulated genes are enriched in gap junction trafficking and regulation, copi-independent golgi-to-ER retrograde traffic, the post-chaperonin tubulin folding pathway, G2/M transition, translocation of GLUT4 to the plasma membrane, the recycling pathway of L1 reactome and immune system. All these biological functions have been previously reported to be associated with aging [79]. Furthermore, we compared these upregulated and downregulated genes with those in the GenAge database [80], which comprises validated genes involved in human aging. Of these genes, six upregulated genes and five downregulated genes are reported in the GenAge database, indicating significant enrichment (Fisher's Exact test *P*-values were 8.66 × 10^−3^ and 4.62 × 10^−3^, respectively) (Fig. S10g). For example, HIF1A is a TF that responds to oxidative stress and oxygen. Previous studies demonstrate that high *HIF1A* expression in microglia participates in the neuroinflammation aging process [81]. Our data show that a new TAD boundary is formed at the PY20 stage, and the interaction between *HIF1A* and its associated CREs significantly increases (Fig. 6d and Fig. S10h). As a result, *HIF1A* is largely upregulated at the PY20 stage, which is associated with brain aging. Taken together, the increase of short-range interaction or decrease of long-range interaction is associated with brain aging.

We also examined the influence of the decrease in long-range interaction. Our results showed that 659 genes lose the enhancer–promoter (E–P) interaction and are downregulated at the PY20 stage compared to PY4.5 stage (Fig. S10i). These genes are enriched in the function of neurotransmitter secretion and neuron projection morphogenesis (Fig. S10i). This suggests that loss of E–P interaction during aging may affect neuronal function. Consistently, the active epigenetic signals of these genes decrease at the PY20 stage compared to the PY4.5 stage, while the H3K27me3 signals increase (Fig. S10j). We further investigated the functions of the genes that lose their E–P interaction at the PY20 stage compared to the PY4.5 stage. The enrichment of these genes in the genes associated with human neuronal diseases was evaluated. The results show that these genes are significantly enriched in the gene sets associated with SCZ, dementia and Alzheimer's disease (Fig. 6e). These results provide a potential explanation for the frequent occurrence of these neuronal diseases in older age groups. The abnormal chromatin structures during aging are involved in the development of neurodegenerative diseases, such as AD.

Previous studies have demonstrated that the activation of transposons is involved in aging [82]. We further checked whether the alternation of epigenetic states of transposons is associated with their activation during brain aging. The data show that the H3K27me3 signals for L1, ERV1 and ERVL-MaLR decrease at the PY20 stage compared to the PY4.5 stage (Fig. 6f). Additionally, our results also confirm that the expression levels of these transposons significantly increase at the PY20 stage (Fig. S4i). Therefore, these findings suggest that the loss of H3K27me3-mediated inhibition is associated with the activation of transposons in the PFC during aging.

Taken together, these data show that the aging process is accompanied with decreased distal interaction and increased local TAD interaction, which results in TAD boundary switch and the abnormal expression of aging related genes.

## DISCUSSION

In this study, we systematically unveiled the chromatin higher-order structure, chromatin accessibility, histone modifications, DNA methylation and gene transcription landscapes of the rhesus monkey PFC from prenatal to aging stages. The dynamics of epigenetic signals during PFC development is associated with stage-specific gene expression, which plays an important role in the regulation of different developmental steps of the PFC. Consistent with previous studies, the chromatin regions of compartments and TADs are stable, while histone modifications are considerably dynamic, such as bivalent histone modifications and H3K27ac modification. It seems that histone modifications exert a more precise function in regulating stage-specific gene expression.

Some features of the brain are different between males and females. For example, brains are larger in males than in females after birth, and are stably 11% larger in adults [83,84]. Previous studies reveal that sex steroid hormones (i.e. androgens, estrogens and progestins) are intimately related to sexual development and further contribute to sexually dimorphic structures, as well as sexual differences in somatic functions and behaviors throughout life [85]. Our data show that at the early fetal stages, the *cis*-modules involved in bRG development are also associated with the genes participating in sex differentiation. Furthermore, the binding motif of estrogen receptor ESR2 is enriched in the L1 retrotransposons at the newly established TAD boundaries during PFC development. This also supports the effect of sex on PFC development. Further investigation is needed to determine whether genes involved in sex differentiation also play a role in bRG development and contribute to the observed differences in the brain between males and females.

Many genes responsible for neuronal cell differentiation and cortex layer specification are pre-configured by bivalent promoters, whereas those involved in establishing the neural network are regulated by super enhancers. This implies that distinct epigenetic regulatory mechanisms can be utilized to regulate different developmental processes. Even so, some genes can be regulated simultaneously by both SE and bivalent promoters. Bivalent modifications are predominantly employed to regulate early developmental events, including cell differentiation. This suggests that the cellular identities and destination within the brain regions of neuronal cells have been predetermined prior to their maturation. Notably, super enhancers have important roles in the establishment of the neural network compared to typical enhancers. This might be attributed to the necessity for coordinated regulation of genes by multiple factors involved in neural network establishment or their heightened activation levels during this process.

Brain aging is a process of chronic homeostasis imbalance [86]. The H3K27me3 signal decreases during aging (Fig. 6f and Fig. S10k). This decrease may contribute to the activation of transposons in aging brains (Fig. S4i). The aberrant activation of transposons results in random transposition of these active transposons and introduces insertion mutations in the genome, which is harmful to the stability of the genome [87]. Consistently, aberrant activation of transposons is detrimental to normal brain functions, which can lead to neuroinflammation and direct neurotoxicity in many neurological disorders [88]. Therefore, both epigenetic and genetic changes in PFC during aging may result in a chronic inflammatory response and persistent activation of the immune system.

We note that genes with stage-specific or dynamic expression are significantly enriched in dynamic TAD boundaries rather than stable TAD boundaries from the E90 stage onwards. The highest enrichment ratio is observed during the transition from the P0 to P4M stage. This observation could be attributed to the fact that the epigenetic signal changes contribute to RNA expression alterations, thereby making dynamic TAD boundaries more influential in gene expression changes than the stable TAD boundaries. Furthermore, epigenetic modifications exhibit the most dynamic changes from the P0 to P4M stage (Fig. 1j). Consequently, this leads to the highest enrichment ratio of genes with stage-specific or dynamic expression in dynamic TAD boundaries during the transition from the P0 to P4M stage. Although the most dynamic changes in epigenetic modification occur from the P0 to P4M stage, gene expression is not dramatically changed. This implies that distinct epigenetic regulatory mechanisms are employed to regulate gene expression between the prenatal and postnatal stages. For instance, the enhancers that regulate or maintain expression of a given gene at the P0 stage may be different from those that regulate expression of the same gene at the P4M stage (Fig. S6a) [89]. Furthermore, different types of epigenetic signals can be used to repress gene expression between prenatal and postnatal stages (Fig. 1k). These may contribute to the dramatic changes in epigenetic signals but no significant change in gene expression between the P0 and P4M stages.

SCZ is believed to arise from abnormalities in multiple genes [90]. *DBN1* is a gene associated with SCZ. Our study identified a CRE regulating the expression of *DBN1* at early developmental stages. Knockout of this CRE leads to defective neuronal proliferation and synaptic neuron differentiation in human cortical organoids. The compromised neuronal proliferation and formation of neuronal network formation during the embryonic stage may represent one of the primary defects underlying the pathogenesis of SCZ. Out of the 63 genes harboring SEs with SNPs associated with SCZ, 20 of these genes have not previously been linked to SCZ according to existing reports (Fig. 4e). Future research could explore the roles of these genes in establishing neuronal networks and contributing to the development of SCZ. These results suggest that certain mutations acquired during the early stages of development might involve genes directly related to the onset of SCZ. Consistently, a previous study revealed that the genes harboring damaging *de novo* mutations identified in SCZ patients were involved in neurogenesis in the fetal PFC [91]. These findings support the theory that disruptions of fetal neurodevelopment are critical to the pathophysiology of SCZ. Alternatively, these mutations, together with additional rare somatic mutations introduced later in adulthood [92], may be associated with the occurrence of SCZ.

The genes linked to the *cis*-element module associated with bRG are notably enriched in evolutionarily young genes. Given that bRG cells are recognized as the major cells contributing to the neuronal expansion in primate brains, this cell type is evolutionarily young. The *cis*-elements regulating bRG may still be undergoing natural selection. This selection process likely involves both positive selection, which introduces mutations to enhance neuronal expansion further, and negative selection, which acts to prevent excessive mutations in these *cis*-elements. We found that the binding motifs of TFs such as CREB5, ETV5 and SOX9 are more enriched in the bRG-associated *cis*-element module, while the binding motifs of ZNF423, TEAD2, TGIF1 and TCF7L1 are more enriched in the aRG-associated *cis*-element module (Fig. 2e). The function of these TFs in the development of bRG and aRG can be explored in the future.

We have observed an increase in short-range interactions during aging, coinciding with a decrease in CTCF signals at TAD boundaries. This loss of chromatin insulation function may lead to a more compact chromatin structure, thereby promoting increased short-range interactions and abnormal gene expression. The reduction in CTCF binding at TAD boundaries could be attributed to some repressive epigenetic signals acquired during aging, such as H3K27me3, which may block the binding motifs of CTCF.

Our data indicate that the BTA genes at the PY4.5 stage are enriched in M13 module for olfactory receptor hub genes. This suggests that the PFC may be influenced by olfactory information at the adult stage. This finding is consistent with previous studies that have provided evidence supporting the influence of olfactory information on the network of the PFC in mice [93].

Our results reveal that DNA methylation replaces H3K27me3 to repress the expression of some genes, especially at the postnatal stage. A potential explanation of this mechanism transition could be that H3K27me3 repression is more flexible, as it can be erased or established during development, while DNA methylation seems to be a more stable repressive signal, which can maintain gene repression over a lifetime.

Single-cell sequencing technologies have become prevalent in brain genomics research. A limitation of our study is that the bulk sequencing methods used cannot account for the heterogeneity of neural cells in the PFC, but only address the dynamics of epigenetic signals across different developmental stages and aging. In the future, employing single-cell sequencing technologies to investigate the dynamics of epigenetic signals for different types of neural cells during PFC development and aging can provide further insights into the regulation of PFC development.

In summary, our study provides a valuable resource for understanding the epigenetic regulatory mechanisms of PFC development in primates.

## MATERIALS AND METHODS

### Ethics of animal experiments

All monkey sample collection and other experimental procedures were approved by the Ethics Committee of the Institute of Zoology and Kunming Institute of Zoology, Chinese Academy of Sciences (IACUC18027) and the Ethics Committee of the Beijing Institute of Genomics (2019A019). All the experiments in this study were in compliance with these relevant ethical regulations. No statistical methods were used to predetermine the sample size.

### Rhesus monkey PFC collection

Anatomical parcellation at each time point relies on previous research conducted on monkey and human brain development [29,41–43]. The study of rhesus monkey embryos at embryo day 50 (E50), E90, birth, 4 months old, 4.5 years old and 20 years old were approved by the Ethics Committee of the Kunming Institute of Zoology, Chinese Academy of Sciences, Kunming, China.

### Sequencing library preparation

The libraries of ChIP-seq, DNase-seq, Hi-C and whole genome bisulfite sequencing were carried out as described [73,94–97]. Total RNA libraries were prepared by using NEBNext Ultra II Directional RNA Library Prep Kit for Illumina (NEB, E7765s). The libraries were sequenced on Hiseq X10 with paired-end 150 bp (Illumina).

### Knockout of target gene enhancer

We designed sgRNAs targeting the putative *DBN1* enhancer by using the GPP sgRNA Designer (CRISPick). The two *DBN1* CRE sgRNAs were cloned into HP180-CBH-Cas9-CMV-EGFP/RFP plasmids, respectively. Human H9 embryonic stem cells were transfected with the above plasmids by electroporation. The GFP and RFP double positive cells were sorted into a 96-well plate coated with Matrigel by fluorescence-activated cell sorting (FACS). The *DNB1* CRE KO clones were identified through DNA agarose electrophoresis and sanger sequencing.

### Culture of cortical organoids

Cortical organoids were generated from the H9 cell line by mimicking the biochemical and physical cues of tissue development and homeostasis.

### Immunostaining of human cortical organoids

Organoids were fixed with 4% paraformaldehyde in phosphate buffer solution (PBS) for 1 h at 4°C, cryoprotected in 30% sucrose and embedded in optimal cutting temperature medium. Cryosections (25 μm) were collected on Superfrost slides using a Leica CM3050S cryostat. Rabbit anti-PAX6 (BioLegend, 901301), mouse anti-TUBB3 (BioLegend, 801201), Rabbit anti-SYN1 (Cell Signaling Technology, 5297S) and mouse anti-pH3 (proteintech, 66863-1-Ig) antibodies were used for immunostaining.

## Supplementary Material

nwae213_Supplementary_Files

## Data Availability

The data sets generated in this study have been deposited in the Genome Sequence Archive under the accession number CRA011791 in PRJCA018217. The scripts used in this study have been uploaded to GitHub and can be accessed via the following link: https://github.com/ningchaozky/rheMac8paper.git. Detailed materials and methods are available in the supplementary data.
