# Supplementary material for: Epigenomic landscapes during prefrontal cortex development and aging in rhesus
Source: Natl Sci Rev. 2024 Jun 18;11(8):nwae213. doi: 10.1093/nsr/nwae213 (PMC11342245; doi:10.1093/nsr/nwae213)
Supplement: nwae213_Supplementary_Files [file nwae213_supplementary_files.zip › Supplementary_Materials/Supplementary_Materials.docx]

## Supplementary Methods

## Rhesus monkey PFC collection

The embryos of E50 and E90 were in vitro fertilized and then transplanted into uterus at blastocyst stage. The rhesus embryos at E120 were obtained from Hengshu Bio-Technology company, Sichuan, China. The fetal monkeys were obtained from cesarean surgery. Then they were killed by euthanasia. The sex information of the rhesus embryos or samples was not collected. The embryos generated from natural pregnancy were collected at E120. The stages of rhesus embryos were further confirmed according to the embryo head sizes. The tissues of the prefrontal cortex were dissected into small pieces and stored in liquid nitrogen. For each developmental stage, two embryos were collected.

## ChIP-seq library preparation

ChIP-seq experiment was carried out as describe [1]. The frozen PFC tissues were thawed on ice, then chopped into very small pieces (<1 mm^3^) using two razor blades. More than one million rhesus cortical cells were used for each ChIP-seq library construction. The samples were resuspended in PBS buffer and subjected to centrifugation at 2,500 rcf for 10 min at 4°C. Supernatant was discarded. The cells were cross linked by PBS containing 1% formaldehyde for 15 min at room temperature (RT). Crosslinking was quenched by adding 145 mM glycine for 5 min at room temperature. Cells were then rinsed two times in ice-cold PBS containing complete protease inhibitor cocktail tablets (Roche) and collected by scraping. Cells were pelleted and either stored at -80°C until use or processed immediately. Cell pellets were lysed by RIPA buffer with (140 mM NaCl, 1 mM EDTA, pH 8.0, 1mM PMSF and com­plete protease inhibitor cocktail) for 30 min at 4°C. Sonication was carried out using a Misonix 3000 Sonicator (Misonix) set at a power setting of 7.5 (equivalent to 24 W). 16 cycles of pulses of 30 s with 30 s interval each were delivered at this setting, which resulted in genomic DNA fragments with sizes ranging from 200 bp to 2 kb. Insoluble materials were removed by centrifugation at 16,000 rcf for 10 min at 4 °C. The supernatant was transferred to a new tube. Before immunoprecipitation, a 1/10 volume of the supernatant was taken as the input control, and stored at -20°for subsequent DNA library preparation. 20 μL beads were pre-incubate with 3 μL antibodies in 100 μL RIPA buffer with com­plete protease inhibitor cocktail for 3 hrs at 4°C, and then added in to samples. The antibody incubation was carried out overnight at 4°C. The next day, the beads bound by immune complexes were pelleted and washed twice with each of the following buffers: low-salt buffer (250 mM NaCl, 1 mM EDTA, pH 8.0, 0.2% SDS, 1mM PMSF and com­plete protease inhibitor cocktail), high-salt buffer (250 mM NaCl, 1 mM EDTA, pH 8.0, 0.2% SDS, 1mM PMSF and com­plete protease inhibitor cocktail). In each wash, the beads were incubated with wash buffer for 5 min at 4°C while nutating. The washed beads were then rinsed once with 1× TE buffer (10 mM Tris-HCl, pH 8.0, and 1 mM EDTA). The immunoprecipitated mate­rial was eluted from the beads by adding 100 μL of elution buffer (10 mM Tris-HCl, pH 8.0, 1 mM EDTA, pH 8.0, 10 mM proteinase K and 1% SDS) to each ChIP reaction and incubating the sample at 65°C for 8 hrs with brief vortex. The immunoprecipitated genomic DNA frag­ments and the previously stored input DNA were then extracted by SPRI beads (Beckman Coulter, B23318). NEBNext Ultra II DNA Library Prep Kit for Illumina (NEB, E7645S) was used to construct library which was submitted to the 150 bp pair-end sequencing on the Illumina Hiseq X10 platform. For each sample, over 20 million clean reads were obtained. The primary antibodies anti-H3K4me3 (Abcam, ab8580), anti-H3K27ac (Abcam, ab4729), anti-H3K27me3 (Active Motif, 39155), anti-Pol2 (Abcam, Ab5131), anti-CTCF (Active Motif, 61311) were used for ChIP-seq.

## DNase-seq library preparation

DNase-seq experiment was carried out as describe [2, 3]. And the condition was optimized based on the samples in this paper. Briefly, the frozen tissue was put in 37^o^C water bath for 10 s to unfreeze, then using a scalpel to cut up the tissue on ice. After dissociate the tissue, add 200 µL 0.1% BSA/PBS to resuspend and transferred into 1.7 mL tubes. Centrifuge at 800 rpm for 5 min at 4^o^C, then remove supernatant. Add 180 µL cold lysis buffer (10 mM Tris-HCL pH 7.5, 10 mM NaCl, 3 mM MgCl_2_, 0.1% Triton X-100) to resuspend the pellet by gentle pipetting, and then kept on ice for 30 min. 20 µL diluted DNase I (Roche, 04716728001) was add to final concentration of 80 U/mL and incubated at 37^o^C for exactly 5 min. Reaction was stopped by adding 400 µL of stop buffer (10 mM Tris-HCL pH 7.5, 10 mM NaCl, 0.15% SDS, 10 mM EDTA) containing 200 µg Proteinase K (QIAGEN, 19133) followed by incubation at 55^o^C for 3 hrs. The DNA was extracted by phenol-chloroform (Amresco, 0883) and precipitated by ethanol with 20 µg glycogen (Thermo Fisher, R0551) and 1/10 volume of 3 M NaOAc (Thermo Fisher, R1181) at -80^o^C overnight. Following centrifugation at max speed for 15 min, DNA precipitation was washed with 800 µL ice-cold 70% ethanol, then dissolved in 50 µL TE (2.5 mM Tris-HCl pH 7.5, 0.05 mM EDTA) after air dry. Run DNA on a 2% agarose gel and cut the gel to select 50-100 bp DNA fragments. Purify size selected DNA using Zymoclean Gel DNA Recovery Kit (ZYMO RESEARCH, D4008). NEBNext Ultra II DNA Library Prep Kit for Illumina (NEB, E7645S) was used to construct library. DNA was end repaired and A-tailed by adding 7 µL NEBNext Ultra II End Prep Reaction Buffer and 3 µL NEBNext Ultra II End Prep Enzyme Mix. Samples were incubated in a thermal cycler at 20^o^C for 30 min, 65^o^C for 30 min, and finally cooled to 4^o^C. Adaptor ligation was performed by adding 30 µL NEBNext Ultra II Ligation Master Mix, 1 µL NEBNext Ligation Enhancer, 0.5 µL 200mM ATP and 2.5 µL Y-shaped Illumina Multiplexing Adaptors (15 µM). Samples were thoroughly mixed and incubated at 20^o^C for 30 min. After adaptor ligation, 1.3 volume SPRIselect beads (Beckman Coulter, B23318) were used to purify DNA and 10-13 cycles PCR amplification was performed with NEBNext Ultra II Q5 Master Mix. PCR product was purified with 1.3 volume of SPRIselect beads. The libraries were sequenced on Hiseq X10 with paired-end 150 bp (Illumina).

## Hi-C library preparation

The Hi-C library preparation was carried out as previously described [4]. Samples were fixed with 100 μL of freshly made 1% formaldehyde solution and incubated at room temperature for 10 min. To quench the reaction, 2.5 M glycine solution was added to a final concentration of 0.2 M. Samples were incubated at room temperature for 5 min and then centrifuged for 5 min at 3000 g at 4^o^C. Supernatants were discarded and samples were lysed in 100 μL of ice-cold Hi-C lysis buffer (10 mM Tris-HCl pH 8.0, 10 mM NaCl, 0.2% Igepal CA630) with protease inhibitor cocktail on ice for 15 min. Samples were then centrifuged at 3000 g for 5 min and the supernatants were carefully discarded. Pelleted nuclei were washed once with 100 μL of 1x NEBuffer 2. The supernatants were discarded with 4.5 μL of liquid retained in the tubes, and then 0.5 μL of 5% sodium dodecyl sulfate (SDS) was added. Tubes were gently tapped to mix the pellet and were incubated at 62^o^C for 20 min. After incubation, 14.5 μL of water and 2.5 μL of 10% Triton X-100 were added to quench the SDS. Tubes were gently tapped to mix well, avoiding excessive foaming, and then incubated at 37^o^C for 15 min. 2.5 μL of 10X NEBuffer 2 and 10 U of MboI restriction enzyme (NEB, R0147) were added and chromatin was digested at 37^o^C for 5 hrs. Samples were incubated at 62^o^C for 20 min to inactivate MboI and then cooled to room temperature. To fill in the restriction fragment overhangs and mark the DNA ends with biotin, 5 μL of fill-in master mix (3.75 μL of 0.4 mM biotin-14-dATP, 0.45 μL of 10 mM dCTP/dGTP/dTTP mixture, 0.8 μL of 5 U/μL DNA polymerase I, large fragment) was added. Samples were mixed by pipetting and incubated at 37^o^C for 30 min. Ligation master mix (66.3 μL of water, 12 μL of 10X NEB T4 DNA ligase buffer, 10 μL of 10% Triton X-100, 1.2 μL of 10 mg/ml bovine serum albumin, 1 μL of 400 U/mL T4 DNA ligase) was added and samples were incubated at 16^o^C for 18 hrs. Nuclei were pelleted by centrifugation for 5 min at 3000 g and were washed with 100 μL of 10 mM Tris buffer, pH 8.0. Pellets were resuspended with 50 μL of 10 mM Tris buffer and 2 μL of 20 mg/ml proteinase K and then incubated at 65^o^C for 18 hrs. Proteinase K was inactivated by incubation at 75^o^C for 30 min. To make biotinylated DNA suitable for high-throughput sequencing by Illumina sequencers, DNA samples were sheared to a length of 400 bp. 50 ng of carrier RNA was added and the sheared DNA was transferred to a new PCR tube, then processed with End Repair/dA-Tailing Module (NEB, E7442L) and Ligation Module (NEB, E7445L) following the operation manual. Samples were prepared for biotin pull-down by washing with 50 μL of 10 mg/ml Dynabeads MyOne Streptavidin T1 beads (Life technologies, 65602) in 100 μL of 1X Tween Washing Buffer (1X TWB: 5 mM Tris-HCl, pH 7.5, 0.5 mM EDTA, 1 M NaCl, 0.05% Tween-20). Samples were separated on a magnet and the solution was discarded. Ligation products were mixed with 83.5 μL of 2x Binding Buffer (2x BB: 10 mM Tris-HCl, pH 7.5, 1 mM EDTA, 2 M NaCl) and centrifuged at 10,000 g for 1 min. Beads were resuspended with supernatant and incubated at room temperature for 30 min with rotation to allow the biotinylated DNA bind to the streptavidin beads. Samples were again separated on a magnet and the solution was discarded. Beads were washed by adding 200 μL of 1X TWB and tubes were heated on a Thermomixer at 55^o^C for 2 min with mixing. Beads were repelleted using a magnet and the supernatant was discarded. This washing step was repeated twice. Beads were then resuspended in 100 μL of 10 mM Tris buffer and transferred to a new tube. Beads were repelleted and the buffer discarded. Beads were resuspended in 20 μL of 10 mM Tris buffer and boiled at 98^o^C for 10 min. Beads were again repelleted and the supernatant was retained. The Hi-C library was amplified by 10 cycles of PCR with Q5 master mix (NEB, M0492L) following the operation manual. PCR products were confirmed by analyzing 1 μL of product using the FlashGelTM System (Lonza, 57063). PCR was continued with additional cycles until bright DNA bands were seen. A bottle of Agencourt AMPure XP beads (Beckman Coulter, A63881) was warmed to room temperature and gently shaken to resuspend the magnetic beads. 100 μL of beads were added to the 200 μL of diluted PCR product (0.5X volumes). Samples were mixed by pipetting and incubated at room temperature for 10 min. Beads were pelleted on a magnet and the clear solution was transferred to a new tube. Another 30 μL of beads were added to the clear solution (0.65X volume), mixed by pipetting, and incubated at room temperature for 10 min. The beads were kept on the magnet, and washed twice with 200 μL of 70% ethanol (freshly made) without mixing. Ethanol was then completely removed. Beads were left on the magnet for 5 min to allow the remaining ethanol to evaporate. DNA was eluted within 20 μL of ddH_2_O. DNA was then quantified and sequenced using an Illumina sequencing platform.

## Whole genome bisulfite sequencing (WGBS) library preparation

WGBS library preparation was carried out as described in a previous study [5]. The genomic DNA of PFC samples was extracted by using QIAamp DNA Mini Kit (QIAGEN) following its user manual. Genomic DNA spiked with 0.5% unmethylated Lambda DNA (Promega) was sonicated into 100-500bp DNA fragments. The fragmented DNA was then subjected to DNA library preparation by using NEBNext Ultra II DNA Library Prep Kit for Illumina (NEB, E7645S). After adaptor ligation, 1x volume of SPRIselect beads were used to purify DNA. Bisulfite conversion was performed using the EZ DNA methylation Gold kit (Zymo Research) according to the manufactory instruction. Bisulfite-treated DNA was then amplified with KAPA HiFi HotStart Uracil+ ReadyMix. Amplified DNA was purified by using 0.8x volume of SPRIselect beads.

## Total RNA-seq library preparation

RNA was extracted using Quick-RNA MicroPrep Kit (ZYMO RESEARCH, R1050) according to the manufactory’s instruction. Then ribosome RNA was removed using NEBNext rRNA Depletion Kit (NEB, E6310). NEBNext Ultra II Directional RNA Library Prep Kit for Illumina (NEB, E7765s) was used to construct library according to the manufactory’s instruction. The libraries were sequenced on Hiseq X10 with paired-end 150 bp (Illumina).

## Knockout of target gene enhancer

We designed sgRNAs targeting the putative *DBN1* enhancer by using the GPP sgRNA Designer (CRISPick) (https://portals.broadinstitute.org/gppx/crispick/public) and then selected the sgRNA sequences with maximum on-target activity and minimum off-target effects of CRISPR-Cas9. The sgRNA sequences were as follows: *DBN1* CRE sgRNA1: ACC AGC TACTCTCCAAGCCG, *DBN1* CRE sgRNA2: GAGAATGGGGAACTTTACTG. The two *DBN1* CRE sgRNAs were cloned into HP180-CBH-Cas9-CMV-EGFP and HP180-CBH-Cas9-CMV-RFP plasmids, respectively. The human H9 embryonic stem cells used in this study were purchased from ATCC. They were tested negative for mycoplasma contamination. In addition, the H9 embryonic stem cells were validated by their morphology, gene expression patterns and organoid formation ability. Human H9 embryonic stem cells were transfected with the above plasmids by electroporation using a Lonza AMAXA 4D-Nucleofector. The transfected human H9 embryonic stem cells were cultured for two days. Then the GFP and RFP double positive cells were sorted into a 96-well plate coated with Matrigel by fluorescence-activated cell sorting (FACS) (one cell per well). The genomic DNA of each clone was extracted to examine the *DBN1* enhancer knockout effect by PCR using the following primers:

*DBN1* CRE forward primer: TGGGCCTTTCTCTACGGCTGC;

*DBN1* CRE reverse primer: TTGCTGAGGTAGCAGACAACCCC.

The *DBN1* enhancer knockout clones were further validated by sequencing. We also assessed the off-target potential of these sgRNAs. The potential off-target sites for each sgRNAs were predicted using CRISPOR v5.01. The top 5 potential off-target sites of each sgRNA were amplified using genomic DNA of the *DBN1* CRE KO cells (Supplementary Table S13). The amplified DNA fragments were sequenced via Sanger sequencing.

## Culture of cortical organoid

Organoids have been generated from H9 cell line by mimicking the biochemical and physical cues of tissue development and homeostasis[6]. Human H9 embryonic stem cells were maintained in Essential 8 Medium (A1517001, Gibco) on 6-well plates coated with Matrigel (354277, Corning). On day 0, we dissociated the target cell colonies into single cells with Accutase (A1110501, Gibco) and suspended them in 100 cells/μL in KSR medium: DMEM/F-12 (11320082, Gibco), 20% KSR (A3181502, Gibco), 2 mM GlutaMax-I (35050061, Gibco), 0.1mM NEAA (11140076, Gibco), 0.1mM beta-mercaptoethanol (21985023, Gibco) with freshly added 10 μM SB431542(1614, TOCRIS), 0.1 μM LDN-193189 (6032, TOCRIS), 3 μM endo-IWR1 (3532, TOCRIS). Then we transferred the cells to 96-well V-bottom plates. We replaced half of the medium with fresh medium once every day until day 18. On day 18, the medium was replaced with neural induction medium containing DMEM/F12, 1:100 N2 supplement (17502048, Gibco), 2mM GlutaMax-I, 0.1mM NEAA, and 0.1 μM beta-mercaptoethanol, and organoids were transferred to 24-well low-cell-adhesion plates. Half of the medium was replaced on alternate days.

## Immunostaining of human cortical organoids

Organoids were fixed with 4% paraformaldehyde in PBS for 1 h at 4 °C, cryoprotected in 30% sucrose and embedded in optimal cutting temperature medium. Cryosections (25 μm) were collected on Superfrost slides using a Leica CM3050S cryostat. The slices were blocked with 10% donkey serum in PBS with 0.1% Triton X-100 at room temperature for 1 h, and then incubated with primary antibodies at 4 °C overnight. Rabbit anti-PAX6 (901301, BioLegend, 1:200 dilution with blocking buffer), mouse anti-TUBB3 (801201, BioLegend, 1:200 dilution with blocking buffer), Rabbit anti-SYN1 (Cell Signaling Technology, 5297S), and mouse anti-pH3 (66863-1-Ig, proteintech, 1:200 dilution with blocking buffer) antibodies were used. After washing three times for 5 min each with washing buffer (0.1% Triton X-100 in PBS) at room temperature, the slides were incubated with Alexa Fluor 488 conjugated donkey anti-rabbit (ab150073, Abcam, 1:200 dilution with blocking buffer) and Alexa Fluor 594 conjugated donkey anti-mouse secondary antibodies (ab150108, Abcam, 1:200 dilution with blocking buffer) at room temperature for 1 h. After washing five times for 5 min each with washing buffer (0.1% Triton X-100 in PBS) at room temperature, the slides were mounted with ProLong Gold Antifade Reagent with DAPI (8961 S, Cell Signaling Technology) for imaging. Images were collected using an Olympus FV3000 confocal microscope.

## ChIP-seq and DNase-seq data analysis

Sequencing data in gzipped fastq file format were unzipped. Low quality bases were trimmed by Trimmomatic with default parameters. Then, sequencing reads were aligned to rhesus genome (rheMac8) using the Burrows-Wheeler Aligner (bwa-0.7.17) with default settings. The uniquely mapped reads were extracted by using Samtools (v1.3.1). The PCR duplicates were removed by picard tool. The numbers of peaks called by the Model base peak-calling algorithm (macs2) [7] and the hotspot algorithm [8] were influenced by sequencing depth. To mitigate this influence, we randomly extracted 35 million reads from each ChIP-seq data set, and 20 million reads from each DNase-seq data set for subsequent analysis. For the DNase-seq libraries, nearly saturation for sequencing was observed with 20 million uniquely mapped reads. In contrast, H3K27me3 and Pol2 signals exhibit broad domain patterns across the genome. As a result, a larger total of 35 million reads were utilized to call peaks in the ChIP-seq data. The ChIP-seq peaks were called by using MACS2-2.2.4 with the following parameters: -f BAM -c Input.bam -g 2.6e9 -m 5 50 -p 1e-3 -B --SPMR --fix-bimodal --broad --nolambda. The ChIP-seq data of input DNA were used as control data for peak calling. The DHSs were called by using hotspot algorithm. Bam files were further transformed to bdg format. Signal at single base pair resolution was obtained by using MACS2 bdgcmp tool. The published ChIP-seq datasets were used for comparison, including human ChIP-seq data ENCFF266SRM from ENCODE project, CTCF ChIP-seq data from GSA: CRA001934[9], H3K4me3 and H3K27ac ChIP-seq data from GSE67978 [10]. To evaluate whether the ChIP-seq peaks or DHSs were enriched in some types of genomic regions, such as promoters, gene body regions, we utilized the annotatePeaks.pl script within homer software by using a customized genomic annotation file and default parameters. If *p* value < 0.05, it means that the ChIP-seq peaks or DHSs is enriched in the specified types of genomic regions.

## Hi-C data processing, mapping, and ICE normalization

The Hi-C data analysis was carried out as previously reported [4]. For Hi-C data, we first trimmed the raw data to remove adaptor sequences and low-quality reads[11]. Then, we mapped the paired-end Hi-C reads to the mouse genome (rheMac8) using HiCUP[12] pipeline v.0.5.7 with parameters (–longest 800 –shortest 150). After HiCUP mapping, we discarded the same fragment reads, re-ligation reads, and continuous reads. Unique mapped reads (mapping quality q > 10)[13] separated by a genomic distance greater than 10 kb were used for subsequent analysis. PCR duplication was removed by Picard Tools[14]. We generated the raw contact matrices at the binning resolution of 10kb, 40 kb, 100 kb, and 250 kb. The ICE [15] normalization was applied to remove bias in the raw matrix using the ICE implemented in HiTC with parameters (–sparse.filter 0.02)[16]. To compare the interaction frequencies among different development stages, interaction matrices should be normalized to remove the effect of sequencing depths at different stages. Thus, each row sum of interaction Iij (*interaction between locus i and locus j*) in the normalized matrix was scaled to 1,0000.

## Pearson correlation coefficients analysis

To validate the reproducibility of our Hi-C data, we used a method described in previous study [4]. We calculated the Pearson correlation coefficients (PCC) between two libraries as follows: The set of all possible interactions, Iij, for two libraries, A and B, were correlated by comparing each point’s interaction profiles in normalized interaction matrix, IA, from library A with the same point, IB, from library B. Because the interaction matrix is highly skewed toward proximal interactions, we restricted the interaction to a maximum distance of 4 Mb between points i and j. We used R functions to calculate the PCC between the two vectors of all points in IA and IB. R package ape was used for sample clustering with 1,000 bootstraps [17] (Supplementary Figure 1b).

To assess the correlation between any two sets of epigenome data, the rhesus genome was binned to 100 bp size for each bin. The average signal of each 100 bp bin for each epigenetic signal was calculated. The paired PCC for the signal of 100 bp bins were calculated.

## TAD signal calculation and relative TAD signal variance

As described in previous study[4], to calculate the dynamics of TAD structures, we calculated TAD signal. The TAD signal value can indicate the strength of the TAD structure. We used intra-chromosomal maps at 10 kb resolution for each stage. The TAD signal was calculated as the log_2_ ratio of the number of corrected upstream-to-downstream interactions within a 2 Mb region. If a region contained less than 10 counts within a 2 Mb distance, the region was filtered. TAD signal variance was only calculated for the loci without filtered-out upstream and downstream regions within a 2 Mb region. Comparisons between different stages were performed only for the bins shared by all of the different stages. Relative TAD signal variance was calculated relative to the minimum TAD signal variance in zygotes. The Wilcoxon test was applied to test statistical significance. To determine the effect of sequencing depth on the TAD signal variance, we performed a downsampling analysis by a downsampling factor of 0.75. Variance due to sampling noise was confirmed to be proportional to 1/reads.

## Insulation score analysis

As described in previous study [4], insulation score can be used to calculate TADs[18]. We used the public code to calculate the insulation score on Github (*matrix2insulation.pl*, https://github.com/dekkerlab/giorgetti-nature-2016). To calculate the insulation score of each bin in the 40 kb, the average number of interactions that occurred across each bin was calculated. This can be visualized by sliding a 480 kb x 480 kb square along the matrix diagonal. The IQR mean signal within the square was then assigned to the 40 kb diagonal bin. This procedure was then repeated for all 480 kb diagonal bins. The insulation score was normalized relative to all of the insulation scores across each chromosome by calculating the log_2_ ratio of each bin’s insulation score versus the mean of all insulation scores. Valleys/minima along the normalized insulation score vector represent the loci of reduced Hi-C interactions that occur across the bin. These valleys/minima are interpreted as TAD boundaries or areas of high local insulation. We compared the insulation score at different stages. We used the Kruskal-Wallis test followed by Dunn’s multiple comparison test to test statistical significance.

To evaluate whether a given set of genes was significantly enriched in dynamic TAD boundaries compared to stable TAD boundaries, we calculated the enrichment ratio using the following equation:

$$Enrichment Ratio=\frac{\frac{number of genes with stage specific or dynamic expression in dynamic TAD boundaries}{number of genes with stage specific or dynamic expression in stable TAD boundaries}}{\frac{number of randomly selected genes in dynamic TAD boundaries}{number of randomly selected genes in stable TAD boundaries}}$$

The significance of the enrichment was assessed using Fisher’s Exact test. A significant enrichment is denoted by a P value < 0.05.

## A/B Compartment PC1 value and status switch

As described [4], we used HOMER [19] software with parameters (-res 25,000 -superRes 100,000) to obtain the PC1 value and A/B compartment status. We used a sliding window approach with a bin size of 100 kb and a step size of 25 kb to generate an observed/expected matrix. The observed interaction was calculated as the sum of all observed interactions of the 25 kb bins making up the larger 100 kb bin. Similarly, the expected interaction was calculated as the sum of the expected interactions of each of the 25 kb bins making up the larger 100 kb bin. This value was used to generate the observed/expected value. PC1 for each chromosome was used to identify regions of the genome as belonging to either the A or B compartment. The direction of the Eigen values is arbitrary, and therefore positive values were set to ‘A’ and negative values were set to ‘B’ based on their association with gene density. We identified regions with statistically significant variability in PC1 values across all developmental stages using ANOVA with p value < 0.05 as A/B compartment switched regions.

To evaluate whether a specified epigenetic or protein binding signal was enriched in Compartment A relative to Compartment B, we compared the ratio of the number of signal peaks in Compartment A to that in Compartment B, with the ratio of the number of randomly selected regions in Compartment A to that in Compartment B. The randomly selected regions match the number and lengths of the epigenetic signal peaks analyzed. Then, the ratio of these two ratios can represent the enrichment of the analyzed signal peaks in Compartment A relative to Compartment B. Fisher’s Exact test was used to evaluated the significance of enrichment. A significant enrichment is defined by a P value < 0.05. The equation to calculate the enrichment is shown as follows:

Enrichment = $\frac{\frac{Number of peaks in Compartment A}{Number of peaks in Compartment B}}{\frac{Number of random regions in Compartment A}{Number of random regions in Compartment B}}$

## Tendency of genes to TAD boundary

The tendency of a given gene to TAD boundary was calculated by the ratio of the distance of this gene to the center of the TAD to the distance of this gene to the TAD boundary. The larger the value, the greater the tendency of the gene to TAD boundary. If a gene is located at a TAD boundary, its tendency value was assigned as the maximum tendency value plus one standard deviation.

## RNA-seq data analysis

Low quality bases were trimmed by Trimmomatic with default parameters. Paired-end reads were aligned to rheMac8 genome by hisat v2.0.4 with parameter “-dta-cufflinks”[20]. We used Cufflinks v2.2.1[21] to calculate the Fragments Per Kilobase of transcript per Million mapped reads (FPKM) of each gene. The Pearson correlation coefficient of gene expression between two replicates was calculated. Then we merged the alignment files from two replicates of each stage, and used Cufflinks to recalculate the FPKM of each gene, representing the expression level of each gene. The genome reference files were downloaded from Ensembl. If a gene had multiple transcripts, we only kept the transcript which harbored the largest FPKM value among all developmental stages for further analysis. The genes with FPKM < 1 were considered as the genes that were not expressed. RNA-seq tracks visualized in UCSC Genome Browser were generated by bamCoverage in Deeptools2.0 suit with parameter “—normalizeUsing RPKM”.

To analyze the expression of transposons, paired-end reads of RNA-seq data were aligned to the rhesus reference genome rheMac8 using HISAT2 v2.0.4 with parameters “-k 1 --dta-cufflinks --rna-strandedness RF”. For multi-mapped reads, only one hit was retained. A published work reports that reporting randomly one hit or weighting multi-mapped reads with the number of hits give rise to the same estimation[22]. The transposon annotation file, “rmsk.txt.gz”, was downloaded from the UCSC genome annotation database for rheMac8. To exclude the impact of transcribed mRNA including both un-spliced and spliced RNA, the transposons located in exons and introns were all excluded from the transposon annotation list. The DNA transposons were also removed from the transposon annotation list. We used Cufflinks v2.2.1 to calculate the Fragments Per Kilobase of transcript per Million mapped reads (FPKM) of each transposon. The expression level of each transposon subfamily was calculated by averaging the levels of all transposons within this subfamily.

## Correlation of epigenetic signals for genomic regions located intra-TADs or inter-TADs

We analyzed the epigenetic signals, including DNase, H3K27ac, H3K4me3, H3K27me3 and Pol2. To clarify whether boundary of TAD restricts slope of modifications, we analyzed the correlation of modifications intra-TADs or inter-TADs at different development stages. First, we equally divided each TAD into ten bins. Then, we calculated the correlation of signals between each pair of two bins within TAD or between a bin within a TAD and the other bin in the upstream TAD or the downstream TAD. Correlations from bins within the same TAD were calculated and referred to intra-TAD correlation. Correlations from bins within different TADs were calculated and referred to inter-TAD correlation.

## WGBS data analysis

Low quality reads were removed by Trimmomatic v0.33. The filtered reads were aligned to reference genomes by using Bismark v0.24.0 with default parameters [23]. PCR duplicates were removed and the methylation level of each CpG site was calculated by using Bismark.

## Identification of dynamically expressed genes

To quantitatively measure the dynamics of gene expression, we calculated the entropy score for each gene by using a previously described method [24]. We first normalized the expression levels (log2 FPKM) by the quantile normalization method among different developmental stages. Then we calculated the entropy score for each gene. We regard the genes with entropy scores < 4.56 as stage-specific expressed genes.

## Gene ontology (GO) analysis

GO analysis was performed by using kobas tool with default parameters[25]. *P*-values were calculated based on the accumulative hypergeometric distribution and *q*-values were the multiple test adjusted *p* values and multiple testing was performed with the Banjamini-Hochberg method.

## Calculation of gene active signal (GAS), stage specificity and cell type enrichment

We profiled H3K4me3, DNase-seq, H3K27ac, Pol2 and H3K27me3 signals in rhesus PFC samples. Among them, the signals of H3K4me3, DHS, H3K27ac and Pol2 represent active transcription states, while the signal of H3K27me3 represents repressive transcription states. In addition, the regions enriched with H3K4me3, DHS and H3K27ac signals are predominantly located in promoters, while those enriched with Pol2 and H3K27me3 signals are mainly found in gene bodies. We attempted to calculate the active transcription signal of each gene at a specified developmental stage by integrating all these profiled signals, and defined it as the gene active signal (GAS). For a specified gene, we used the signals of H3K4me3, DHS and H3K27ac in the promoter, as well as the signals of H3K27me3 and Pol2 in the gene body to calculate GAS. We selected TSS ± 2 kb and gene body ± 5 kb for calculating the signal in promoter and gene body, respectively. To calculate the signal of a particular type of signal, such as H3K4me3, we employed the representative value (*V_bp_*) for each genomic base pair in the bedGraph file generated by MACS2 software, which represents the -log_10_P-value of the H3K4me3 signal. If the promoter of the gene overlaps a H3K4me3 peak at a specified stage, we used all the *V_bp_* in the H3K4me3 peak to calculate the H3K4me3 signal for this gene at that stage using four different methods: max of *V_bp_*, median of *V_bp_*, mean of *V_bp_*, and sum of *V_bp_*, represented as *S_H3K4me3_max_*, *S_H3K4me3_median_*, *S_H3K4me3_mean_*, *S_H3K4me3_sum_*, respectively. If the promoter of the gene does not overlap any H3K4me3 peaks at a specified stage, the H3K4me3 signal for this gene at that stage is assigned to a value of 0. For H3K4me3 signals calculated by different methods, the H3K4me3 signal values of the gene calculated by the same method at different developmental stages from E50 to PY20 were normalized to a range between 0 to 1. At last, we obtained a set of normalized H3K4me3 signal values for a gene at a development stage: [*S_H3K4me3_max_normalized_*, *S_H3K4me3_median_normalized_*, *S_H3K4me3_mean_normalized_*, *S_H3K4me3_sum_normalized_*]. Next, we calculated the correlations between the normalized H3K4me3 signal values [*S_H3K4me3_max_normalized_*, *S_H3K4me3_median_normalized_*, *S_H3K4me3_mean_normalized_*, *S_H3K4me3_sum_normalized_*] and RNA expression levels at different developmental stages during PFC development, respectively. Among *S_H3K4me3_max_normalized_*, *S_H3K4me3_median_normalized_*, *S_H3K4me3_mean_normalized_* and *S_H3K4me3_sum_normalized_*, the one exhibiting the highest correlation coefficient with RNA expression were selected as the normalized H3K4me3 signal for that gene. Employing a similar strategy, we calculated the normalized H3K4me3 signal values for all genes, and calculated the normalized H3K27ac signal values, DHS signal values in promoters for all genes, the normalized H3K27me3 and Pol2 signal in gene bodies for all genes. Finally, the GAS of a gene was calculated by the summing of normalized H3K4me3, H3K27ac, DHS, Pol2 signal values then subtracting the normalized H3K27me3 signal value.

Next, we evaluated whether a gene had a stage-specific GAS. For a gene exhibiting stage-specific GAS, it must meet two specific conditions: (1) the maximum GAS for the gene at all developmental stages must be greater than 0, (2) the ratio of this maximum GAS to the GAS at any other developmental stages must be greater than 2.

To assess the influence of different types of epigenetic signals on stage-specific gene expression, among the stage-specifically expressed genes, we calculated for each epigenetic signal type the number of the genes with both the highest epigenetic signal and the highest gene expression levels at a given stage. This count was denoted as *N_epigenetic_signal_*. A larger N value indicates a more pronounced contribution of the epigenetic signal to stage-specific gene expression. The differential contribution of epigenetic signals to stage-specific gene expression between two distinct types was further assessed using a chi-square test to determine statistical significance.

To assess the enrichment of stage-specifically expressed genes or genes with stage-specific GASs in the marker genes of a specified cell type, the *p*-value was calculated through two methods: CIBERSORTx algorithm [26] and a hypergeometric distribution test. If the results from both methods showed significant enrichment (*p* < 0.05), the more significant enrichment results between the two methods were used for presentation. The marker genes of various types of neural cells were obtained from previously published studies [27, 28]. The published gene lists of marker genes have been consolidated and provided in the Supplementary Table S6. The enrichment ratio was calculated according to following equation:

$$Enrichment ratio=\frac{Number of stage specific genes in a gene list of a specified type of neural cells}{Number of random genes in a gene list of a specified type of neural cells}$$

## Cumulative fractions analysis

We first obtained the gene lists for all genes, protein coding genes, all disease-associated genes, neuronal disease-associated genes, neuronal disease-associated transcription factors. All disease-associated genes were collected from <https://www.disgenet.org/>, <https://www.omim.org/>, and a public database Diseases 2.0 [29]. The neuronal disease-associated genes were collected from <https://www.omim.org> and <https://www.ebi.ac.uk/gwas/>. Among all disease-associated genes, the genes that did not fall under neuronal disease-associated genes were classified as non-neuronal disease-associated genes. A list of published human transcription factors was used to identify the neuronal disease-associated transcription factors [30]. The gene lists for these categories were provided in Supplementary Table S7. Next, we overlapped the genes exhibiting active signal at different stages during PFC development with the genes in the aforementioned gene lists. For the genes with active signal in each gene list, we categorized the genes based on their initial developmental stages when active signal was detected. We then enumerated the number of genes within each category and calculated the proportion of gene count for each category. Subsequently, we computed the cumulative fraction of genes for each developmental stage by summing the proportions of gene counts at the corresponding developmental stage and prior stages. For example, the cumulative fraction at E120 stage was computed by summing gene count proportions at the E50, E90, and E120 stages. Ultimately, we plotted the curve representing these cumulative fractions. The rate of increase in the cumulative fraction can reflect preferences of the developmental stages when the genes establishing active signal for each gene set. A high rate of increase rate indicates that the genes within a given gene set tend to establish active signal at early developmental stages.

## Weighted gene co-expression network analysis (WGCNA) for active signal in promoter region

We used Weighted Gene Co-expression Network Analysis (WGCNA, v1.63) [31] for network analysis using active signal in promoter of protein coding genes. We then estimated the co-expression network and modules using the function blockwiseModule with the following parameters: maxBlockSize = 22000, corType=bicorr, power=16, networkType=signed, deepSplit=2, minModuleSize=50, pamRespectsDendro=F, mergeCutHeight=0.1. The analysis produced a network of 28 modules, plus the grey module of unassigned genes. These modules were further subjected to k-means clustering using R function kmeans (iter.max=1000, nstart =10, algorithm = “Hartigan-Wong”), generating 7 clusters as super-module. Gene ontology item enrichment analysis for genes in each module were performed by using kobas tool.

## Gene age analysis

The gene age classification was performed by using a phylostratigraphic approach [32]. In this method, the gene emergence at different levels of the taxonomic hierarchy in the tree of eukaryotic life was analyzed. The genes of a given genome could be placed into different phylogenetic levels, called phylostrata (PS), according to where their homologues could be found by Blast analysis. Therefore, PS values can reflect the evolutionary ages of genes. The smaller the PS value, the older the gene. Evolutionary age data for primate orthologous genes were obtained from this study [32]. The primate genes are classified into 19 phylogenetic levels from PS1 to PS19, with P1 genes emerging as early as cellular organism and PS19 genes emerging with the appearance of primates during evolution.

## Super enhancer identification

To call super enhancers, we used ROSE algorithm [33] to analyze H3K27ac ChIP-seq data with default parameters except setting STITCHING_DISTANCE = 8k. The enrichment of the SE- and TE-associated genes within the intelligence-associated genes was assessed by using a hypergeometric distribution test, utilizing randomly selected genes as controls. The enrichment P values were adjusted using Benjamini-Hochberg to yield the adjusted P values (FDR).

To compare the enrichment of SNPs in enhancers between TE and SE, considering the influence of enhancer lengths, we recalculated the odds ratio using the following equation:

$$odds ratio=\frac{\frac{Number of SNPs in SEs}{Number of SNPs in TEs}}{\frac{Total lengths of SEs}{Total lengths of TEs}}$$

To further examine the distribution of schizophrenia-associated SNPs within SEs and TEs, we randomly selected a set of 50 single nucleotide sites in the genome. Using Fisher’s exact test, we observed that the distribution of schizophrenia-associated SNPs in SE and TE is significantly different compared to the random scenario. Furthermore, given that the odds ratio is > 1, it indicates a greater enrichment of schizophrenia-associated SNPs within SEs compared to TEs.

## Identification of stage-specific enhancers

To identify stage-specific enhancers, we integrated H3K27ac ChIP-seq, DNase-seq, Hi-C, and CTCF ChIP-seq data. The *cis*-element located in distal regions within 3 Mb both upstream and downstream of transcription start sites but not in promoters, exhibiting signals of H3K27ac, CTCF and DHS, and interacting with promoters were defined as putative enhancers. Next, we identified stage-specific enhancers based on their H3K27ac signal by using similar strategy of identification of stage-specific GAS.

## Linkage disequilibrium score (LDSC) regression analysis

LDSC regression estimates both heritability and confounding biases, such as cryptic relatedness and population stratification, among single-nucleotide polymorphisms (SNPs) by using only summary statistics released from genome-wide association studies (GWAS) [34]. We obtained GWAS summary statistics for Crohns’ disease, education year, intelligence, epilepsy, Alzheimer’s disease, bipolar disorder, schizophrenia, and autism. We first analyzed the heritability of specific neurological disorders, psychiatric disorders and neurobehavioral traits, and applied a stratified LDSC to identify neuronal disease- or neurobehavioral traits-associated loci and genes (P value < 0.05) [35]. We then evaluated the enrichment of the marker genes of different brain cell types in the neuronal disease-associated genes by using hypergeometric distribution test, with randomly selected genes used as control. The stage-specific enhancers were also assessed for the enrichment in neuronal disease associated loci, with randomly selected genomic regions used as control.

## Long range interaction calculated from Hi-C data

We have leveraged Hi-C data to annotate GWAS risk loci. After mapping and filtering the reads, we constructed normalized contact matrices at 40 kb resolution for loop analysis. We detected interaction between promoter of each protein coding gene with each bin $\pm$2 MB from promoter. The cutoff of P-values < 1x10^–10^ was selected as significant promoter-based interactions. The TE- or SE-associated genes were chosen according to the enhancer-promoter interaction detected in the Hi-C data.

## Identification of genes connected to schizophrenia-risk variants

We collected candidate SNPs in the schizophrenia-associated loci (SNPs with *r^2^*  ≥  0.6) with one of the independent significant SNPs (*p-values* < 1x10^–5^) from GWAS catalog. The distal *cis*-regulatory elements overlapping with these schizophrenia-associated SNPs were selected and considered as putative schizophrenia-associated *cis*-elements for further analysis. Putative schizophrenia-risk genes were identified if their promoters had interactions with putative schizophrenia-associated *cis*-elements according to Hi-C data.

## Motif enrichment analysis

We used *fimo* program in MEME suite to perform motif analysis with parameter “--thresh 1e-3”. Multiple motif libraries were used for this analysis, including JASPAR2024_CORE_non-redundant_pfms, HOCOMOCOv10_HUMAN_mono_meme_format, HOCOMOCOv11_core_HUMAN_mono_meme_format, HOCOMOCOv11_full_HUMAN_mono_meme_format HOCOMOCOv9. The results from these libraries were consolidated and sorted based on the p values.

## Statistical analysis

The statistical analysis was performed by R v3.6.1. Pearson’s correlation coefficient was calculated by cor.test function with default parameters. Fisher’s Exact test and a hypergeometric distribution test were used to evaluate gene enrichment. Wilcoxon rank sum test was used for comparison of evolutionary conservation of *cis*-elements, gene densities between A and B compartment. Student’s t test with a two-sided model was used to compare TAD sizes in PFCs at different stages, gene expression level of *DBN1* between *DBN1* CRE KO and control organoid, the number of synaptic connections between *DBN1* CRE KO and control organoid.

## References

1. Wu K, Fan D, Zhao H *et al.* Dynamics of histone acetylation during human early embryogenesis. *Cell Discov*. 2023; **9**(1): 29. doi: 10.1038/s41421-022-00514-y

2. Gao L, Wu K, Liu Z *et al.* Chromatin Accessibility Landscape in Human Early Embryos and Its Association with Evolution. *Cell*. 2018; **173**(1): 248-259.e215. doi: 10.1016/j.cell.2018.02.028

3. Yao X, Lu Z, Feng Z *et al.* Comparison of chromatin accessibility landscapes during early development of prefrontal cortex between rhesus macaque and human. *Nature Communications*. 2022; **13**(1): 3883. doi: 10.1038/s41467-022-31403-3

4. Ke Y, Xu Y, Chen X *et al.* 3D Chromatin Structures of Mature Gametes and Structural Reprogramming during Mammalian Embryogenesis. *Cell*. 2017; **170**(2): 367-381 e320. doi: 10.1016/j.cell.2017.06.029

5. Xu X, Li G, Li C *et al.* Evolutionary transition between invertebrates and vertebrates via methylation reprogramming in embryogenesis. *National Science Review*. 2019; **6**(5): 993-1003. doi: 10.1093/nsr/nwz064

6. Lancaster MA, Knoblich JA. Organogenesis in a dish: modeling development and disease using organoid technologies. *Science (New York, NY)*. 2014; **345**(6194): 1247125. doi: 10.1126/science.1247125

7. Zhang Y, Liu T, Meyer CA *et al.* Model-based Analysis of ChIP-Seq (MACS). *Genome biology*. 2008; **9**(9): R137. doi: 10.1186/gb-2008-9-9-r137

8. John S, Sabo PJ, Thurman RE *et al.* Chromatin accessibility pre-determines glucocorticoid receptor binding patterns. *Nature genetics*. 2011; **43**(3): 264-268. doi: 10.1038/ng.759

9. Luo X, Liu Y, Dang D *et al.* 3D Genome of macaque fetal brain reveals evolutionary innovations during primate corticogenesis. *Cell*. 2021; **184**(3): 723-740 e721. doi: 10.1016/j.cell.2021.01.001

10. Vermunt MW, Tan SC, Castelijns B *et al.* Epigenomic annotation of gene regulatory alterations during evolution of the primate brain. *Nat Neurosci*. 2016; **19**(3): 494-503. doi: 10.1038/nn.4229

11. Bolger AM, Lohse M, Usadel B. Trimmomatic: a flexible trimmer for Illumina sequence data. *Bioinformatics*. 2014; **30**(15): 2114-2120. doi: 10.1093/bioinformatics/btu170

12. Wingett S, Ewels P, Furlan-Magaril M *et al.* HiCUP: pipeline for mapping and processing Hi-C data. *F1000Res*. 2015; **4**: 1310. doi: 10.12688/f1000research.7334.1

13. Li H, Handsaker B, Wysoker A *et al.* The Sequence Alignment/Map format and SAMtools. *Bioinformatics*. 2009; **25**(16): 2078-2079. doi: 10.1093/bioinformatics/btp352

14. DePristo MA, Banks E, Poplin R *et al.* A framework for variation discovery and genotyping using next-generation DNA sequencing data. *Nature genetics*. 2011; **43**(5): 491-498. doi: 10.1038/ng.806

15. Imakaev M, Fudenberg G, McCord RP *et al.* Iterative correction of Hi-C data reveals hallmarks of chromosome organization. *Nature methods*. 2012; **9**(10): 999-1003. doi: 10.1038/nmeth.2148

16. Servant N, Lajoie BR, Nora EP *et al.* HiTC: exploration of high-throughput ‘C’ experiments. *Bioinformatics*. 2012; **28**(21): 2843-2844. doi: 10.1093/bioinformatics/bts521

17. Paradis E, Claude J, Strimmer K. APE: Analyses of Phylogenetics and Evolution in R language. *Bioinformatics*. 2004; **20**(2): 289-290. doi: 10.1093/bioinformatics/btg412

18. Giorgetti L, Lajoie BR, Carter AC *et al.* Structural organization of the inactive X chromosome in the mouse. *Nature*. 2016; **535**(7613): 575-579. doi: 10.1038/nature18589

19. Heinz S, Benner C, Spann N *et al.* Simple Combinations of Lineage-Determining Transcription Factors Prime cis-Regulatory Elements Required for Macrophage and B Cell Identities. *Molecular cell*. 2010; **38**(4): 576-589. doi: <https://doi.org/10.1016/j.molcel.2010.05.004>

20. Kim D, Langmead B, Salzberg SL. HISAT: a fast spliced aligner with low memory requirements. *Nat Methods*. 2015; **12**(4): 357-360. doi: 10.1038/nmeth.3317

21. Trapnell C, Williams BA, Pertea G *et al.* Transcript assembly and quantification by RNA-Seq reveals unannotated transcripts and isoform switching during cell differentiation. *Nature biotechnology*. 2010; **28**(5): 511-515. doi: 10.1038/nbt.1621

22. Teissandier A, Servant N, Barillot E *et al.* Tools and best practices for retrotransposon analysis using high-throughput sequencing data. *Mob DNA*. 2019; **10**: 52. doi: 10.1186/s13100-019-0192-1

23. Krueger F, Andrews SR. Bismark: a flexible aligner and methylation caller for Bisulfite-Seq applications. *Bioinformatics*. 2011; **27**(11): 1571-1572. doi: 10.1093/bioinformatics/btr167

24. Shen Y, Yue F, McCleary DF *et al.* A map of the cis-regulatory sequences in the mouse genome. *Nature*. 2012; **488**(7409): 116-120. doi: 10.1038/nature11243

25. Xie C, Mao X, Huang J *et al.* KOBAS 2.0: a web server for annotation and identification of enriched pathways and diseases. *Nucleic acids research*. 2011; **39**(Web Server issue): W316-322. doi: 10.1093/nar/gkr483

26. Newman AM, Steen CB, Liu CL *et al.* Determining cell type abundance and expression from bulk tissues with digital cytometry. *Nature biotechnology*. 2019; **37**(7): 773-782. doi: 10.1038/s41587-019-0114-2

27. Zhong S, Zhang S, Fan X *et al.* A single-cell RNA-seq survey of the developmental landscape of the human prefrontal cortex. *Nature*. 2018; **555**(7697): 524-528. doi: 10.1038/nature25980

28. Lake BB, Ai R, Kaeser GE *et al.* Neuronal subtypes and diversity revealed by single-nucleus RNA sequencing of the human brain. *Science*. 2016; **352**(6293): 1586-1590. doi: 10.1126/science.aaf1204

29. Grissa D, Junge A, Oprea TI *et al.* Diseases 2.0: a weekly updated database of disease-gene associations from text mining and data integration. *Database (Oxford)*. 2022; **2022**. doi: 10.1093/database/baac019

30. Lambert SA, Jolma A, Campitelli LF *et al.* The Human Transcription Factors. *Cell*. 2018; **172**(4): 650-665. doi: 10.1016/j.cell.2018.01.029

31. Langfelder P, Horvath S. WGCNA: an R package for weighted correlation network analysis. *BMC bioinformatics*. 2008; **9**: 559. doi: 10.1186/1471-2105-9-559

32. Domazet-Loso T, Tautz D. An ancient evolutionary origin of genes associated with human genetic diseases. *Mol Biol Evol*. 2008; **25**(12): 2699-2707. doi: 10.1093/molbev/msn214

33. Whyte WA, Orlando DA, Hnisz D *et al.* Master transcription factors and mediator establish super-enhancers at key cell identity genes. *Cell*. 2013; **153**(2): 307-319. doi: 10.1016/j.cell.2013.03.035

34. Song S, Jiang W, Zhang Y *et al.* Leveraging LD eigenvalue regression to improve the estimation of SNP heritability and confounding inflation. *American journal of human genetics*. 2022; **109**(5): 802-811. doi: 10.1016/j.ajhg.2022.03.013

35. Bulik-Sullivan BK, Loh PR, Finucane HK *et al.* LD Score regression distinguishes confounding from polygenicity in genome-wide association studies. *Nature genetics*. 2015; **47**(3): 291-295. doi: 10.1038/ng.3211

## Supplementary Data Figures, Legends, and Table Titles

**
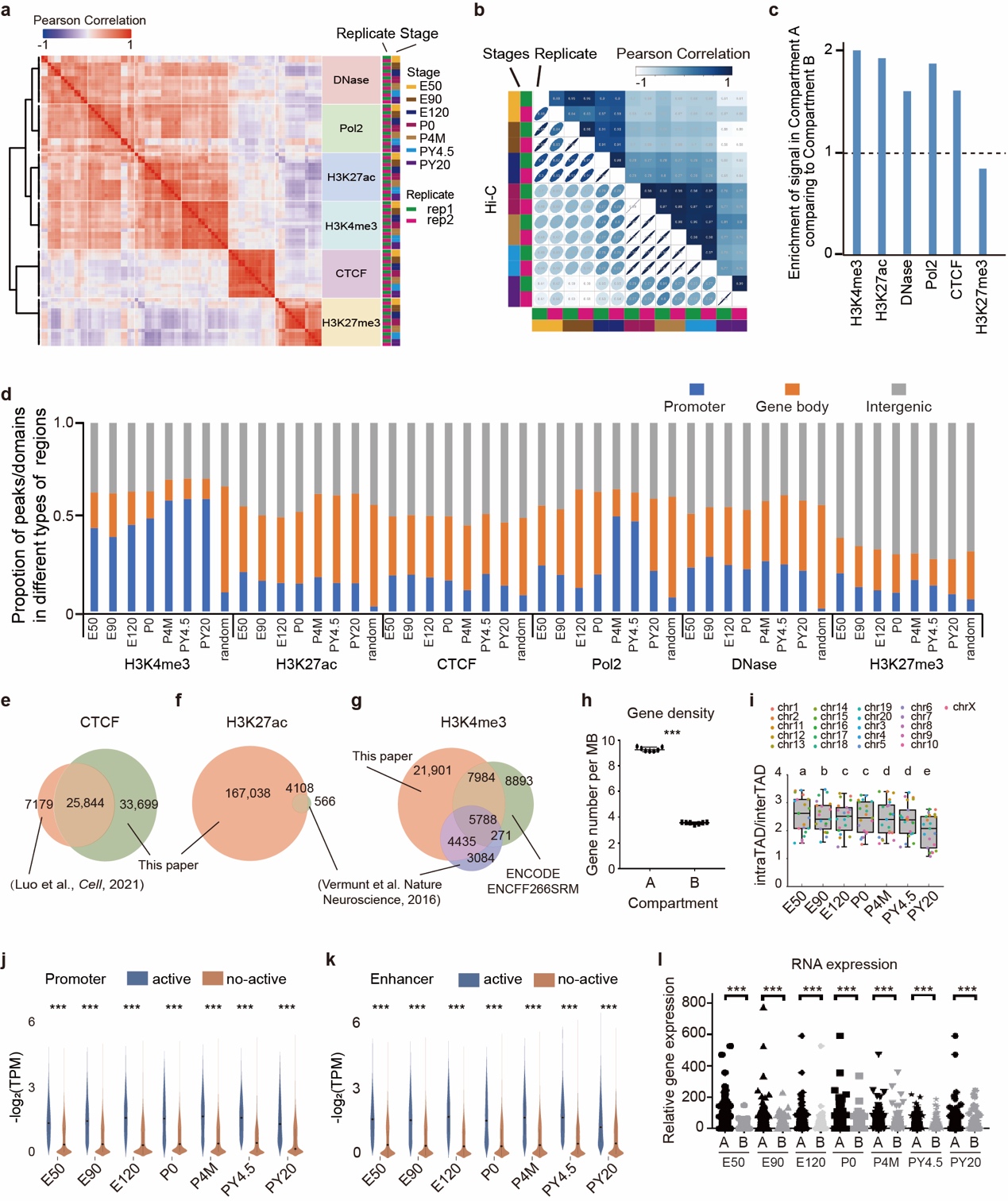
**

## Supplementary Figure 1. Quality validation of epigenome data of PFCs during rhesus development.

**(a)** Correlation among different types of epigenetic signal and protein binding signal in rhesus PFC samples. The hierarchical clustering result of different signal is shown. The whole-genome signal patterns of active markers including H3K4me3, H3K27ac, Pol2 and DHSs are clustered together, which are negatively correlated with that of repressive marker H3K27me3. (**b)** Correlation of Hi-C signal among rhesus PFC samples. **(c)** Bar plot showing the enrichment of different types of epigenetic signals or protein binding signals in Compartment A compared to Compartment B. **(d)** Genomic distribution of each epigenetic and protein binding signal in rhesus genome. The peaks of H3K4me3, CTCF, Pol2 and H3K27ac are higher, and H3K27me3 and DNase lower enriched in TSS regions. **(e)** Overlap of CTCF peaks between this study and published data (Luo et al., *Cell*, 2020). **(f)** Overlap of H3K27ac peaks between this study and published data (Vermunt et al., *Nature Neuroscience*, 2016). **(g)** Overlap of H3K4me3 peaks among this study and published data (Vermunt et al., *Nature Neuroscience*, 2016; ENCODE ENCFF266SRM). **(h)** Gene densities of A compartments and B compartments at all stages. The gene density is calculated as the gene number per million base pairs. Wilcoxon rank sum test is used. *** represents p < 0.001. **(i)** Box and jitter plots showing the ratio of intra-TAD interaction to inter-TAD interaction in each chromosome in rhesus. The characters “a-e” above the boxes are labels for statistically differences. The boxes labeled with different characters means the difference between these datasets is significant. The boxes labeled with the same characters means the difference between these datasets is not significant. For example, the ratios between the E120 and P0 stages (both are labeled with “c”) is not significantly different. **(j-k)** Violin plots comparing the expression levels between genes with and without active signal in their cis-elements including promoters **(j)** and enhancers **(k)**. The genes with active signals at the associated cis-elements show higher expression levels than those without. Wilcoxon rank sum test is used. *** represents *p* < 0.001. TPM, transcripts per million. **(l)** Comparison of expression levels between genes in A compartments and genes in B compartments. Wilcoxon rank sum test is used. *** represents p < 0.001.


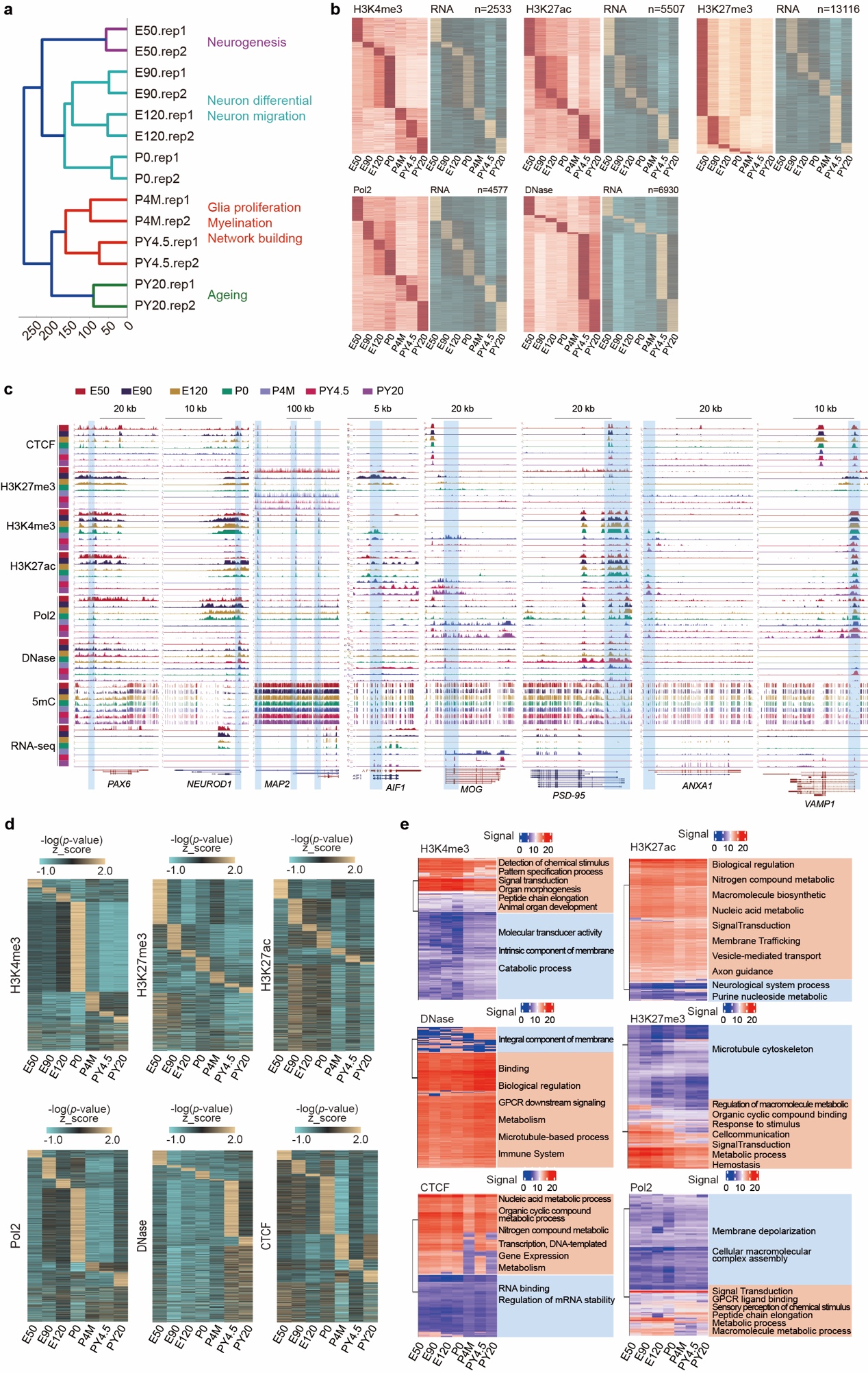


###### Supplementary Figure 2. Stage-specific epigenetic signal of genes and GO analysis of the genes during PFC development.

**(a)** Hierarchical clustering of rhesus PFC samples according to the epigenetic signal. The related developmental events of PFC are shown. **(b)** Heatmaps showing the signal of stage-specific H3K4me3, H3K27ac, H3K27me3, Pol2, DNase-seq, and the expression of associated genes. The number of genes with stage-specific epigenetic signal for each type of epigenetic signal were shown. **(c)** Genome browser view of epigenetic and transcriptional signal of the stage-specifically regulated genes (SRGs). The promoters of these genes are labeled in steel blue shadows. **(d)** Heat map showing the epigenetic and protein binding signals of the genes exhibiting stage-specific and stage-shared epigenetic or protein binding signals for each type of epigenetic or protein binding signal. **(e)** Heat maps showing the epigenetic and protein binding signals of the genes exhibiting stage-common signals for different types of epigenetic signals. For each type of signal, the genes were divided into two clusters. The GO results of each cluster of genes were shown.


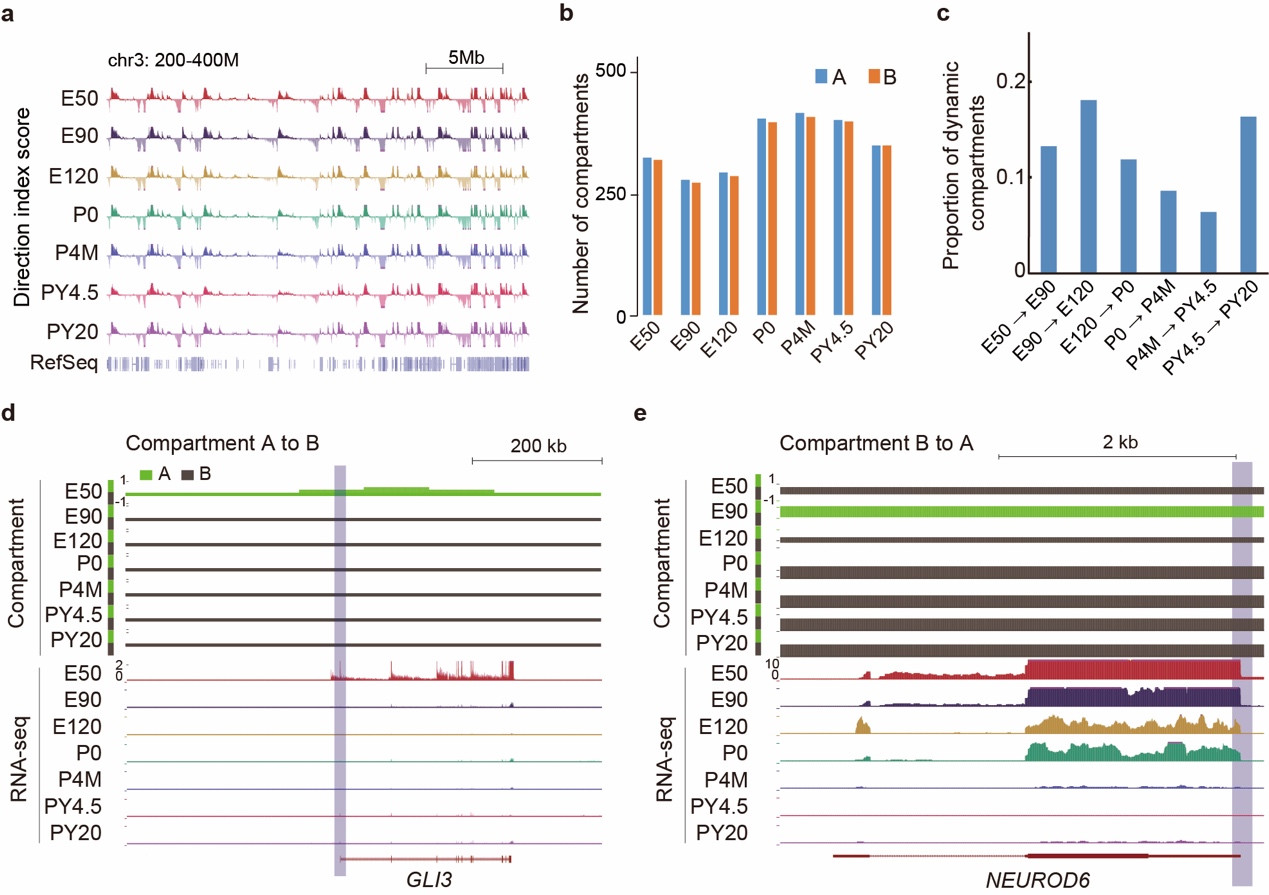


Supplementary Figure 3. The dynamics of chromatin compartment during PFC development.

**(a)** Genome browser view of direction index scores in a genomic region during PFC development. **(b)** The numbers of A and B compartments across developmental stages. **(c)** Bar plot showing the proportion of compartments exhibiting compartment change between adjacent developmental stages. **(d)** Genome browser view of compartment signal and expression pattern of *GLI3*, which can inhibit glia cell fate determination. The promoter region is labeled in a purple shadow. **(e)** Genome browser view of compartment signal and expression pattern of *NEUROD6*, which participates in neuronal differentiation. The promoter region is labeled in a purple shadow.


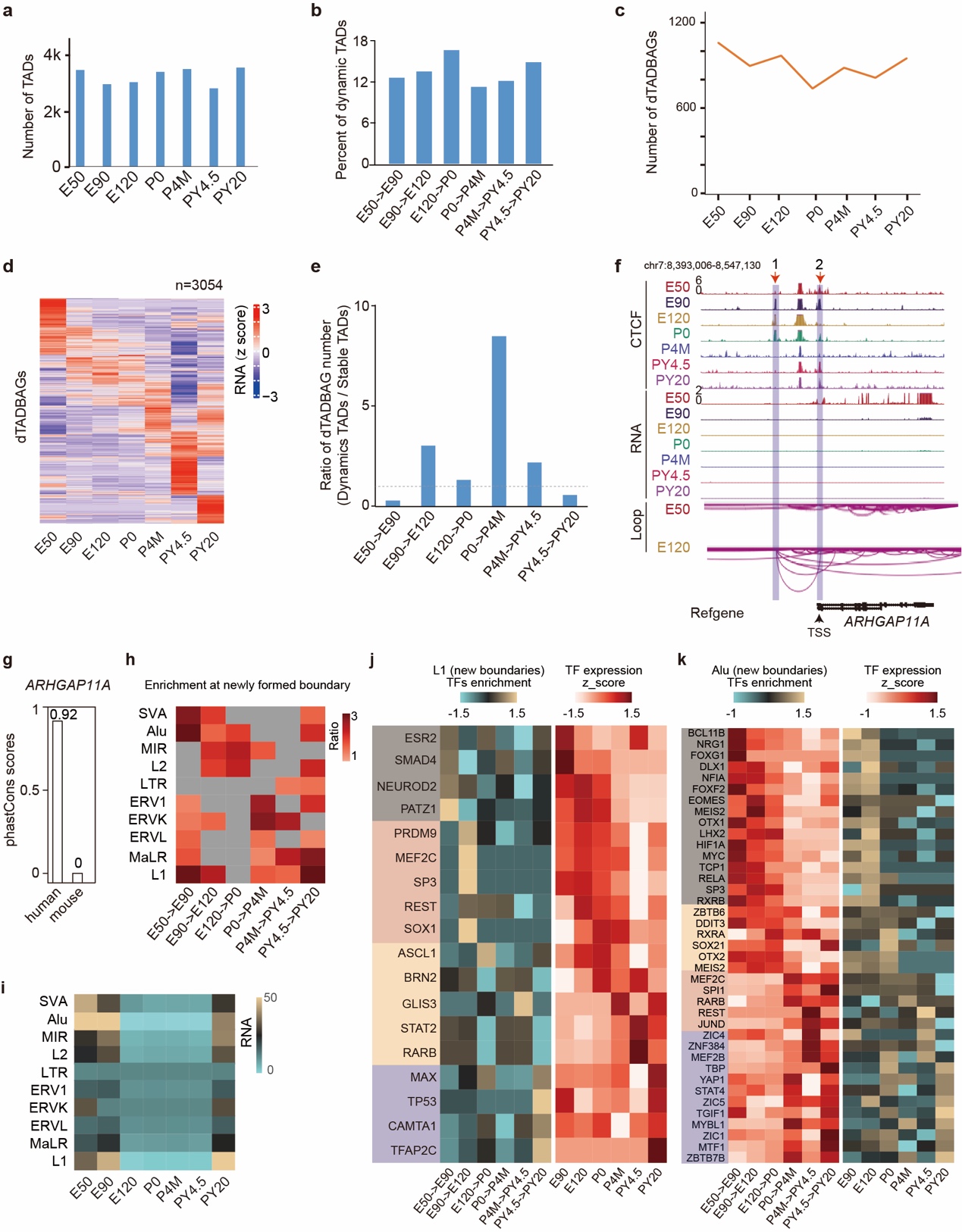


## Supplementary Figure 4. The dynamic of TAD and epigenetic signals at TAD boundaries during PFC development.

**(a)** Bar plot showing TAD numbers in rhesus PFC samples. **(b)** Bar plot showing the percentage of TADs undergoing TAD changes between two adjacent development stages. **(c)** The number dTADBAGs in rhesus PFCs. dTADBAGs, dynamic TAD boundary-associated genes. **(d)** Heat map showing the expression patterns of dTADBAGs which exhibit stage-specific or dynamic expression. The number of the genes is shown. **(e)** Bar plot showing the number ratio of dynamic TADs to stable TADs, between two adjacent developmental stages. **(f)** Genome browser view of CTCF, RNA and loop interaction at *ARHGAP11A* locus. The two CTCF binding sites are labeled by red arrows. **(g)** Bar plot showing the phastCons scores of *ARHGAP11A* between rhesus and human, and between rhesus and mouse. **(h)** The enrichment of transposons in the newly formed TAD boundaries between two adjacent developmental stages. **(i)** Heat map showing expression levels of retrotransposons at different development stages in rhesus PFCs. **(j)** The enrichment of TF binding motifs in L1 retrotransposons located in the newly formed TAD boundaries and the expression of the TFs. **(k)** The enrichment of TF binding motifs in Alu retrotransposons located in the newly formed TAD boundaries and the expression of the TFs.


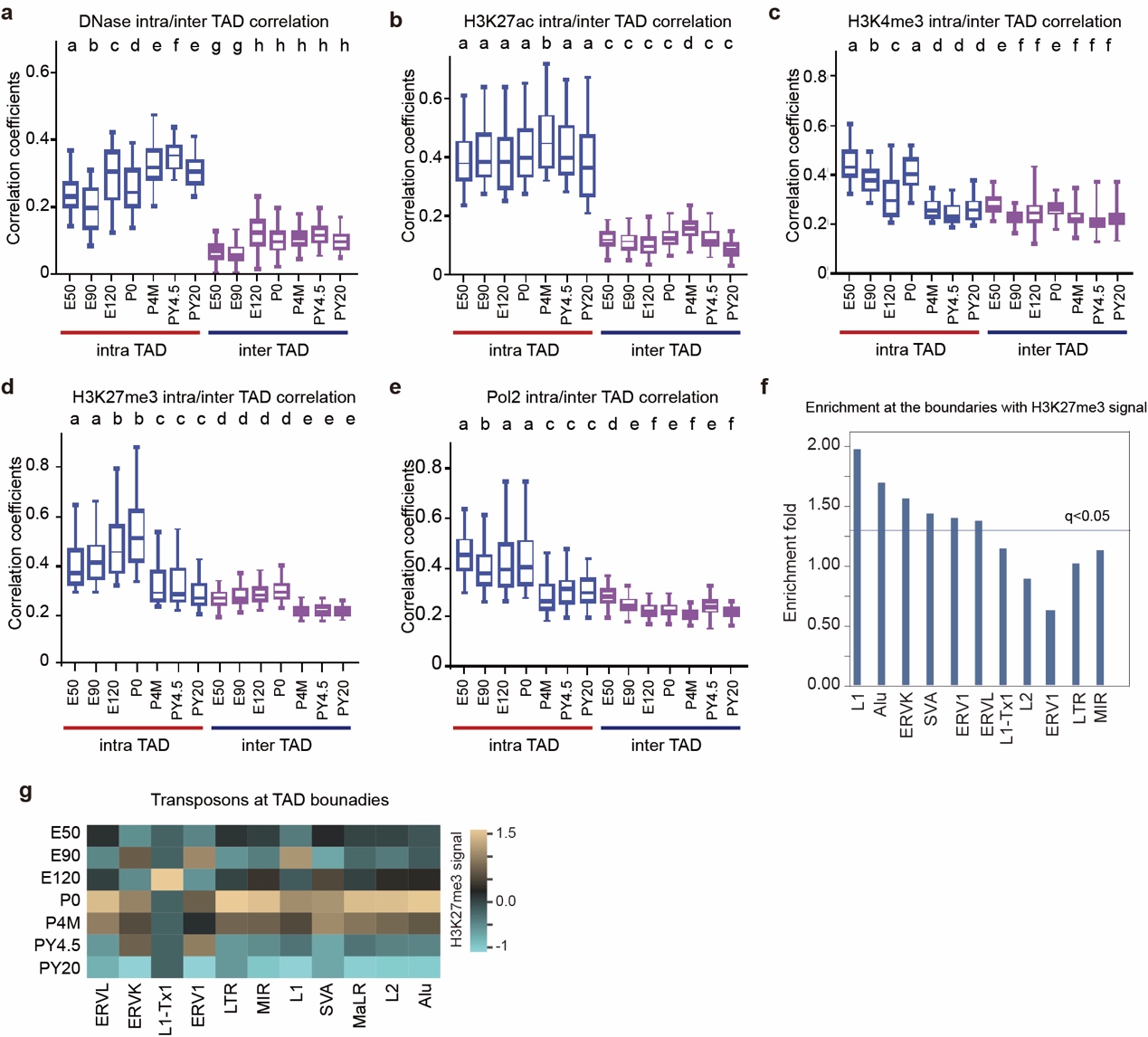


## Supplementary Figure 5. The dynamics of epigenetic signals related with TADs during PFC development.

**(a-e)** Box plots showing intra-TAD signal correlation and inter-TAD signal correlation during PFC development for different epigenetic signal. The characters “a-h” above the boxes are labels for statistically differences. The boxes labeled with different characters means the difference between these datasets is significant. The boxes labeled with the same characters means the difference between these datasets is not significant. **(f)** The enrichment of retrotransposons in the TAD boundaries with enriched H3K27me3 signal. **(g)** The H3K27me3 signals of transposons at TAD boundaries at different developmental stages.


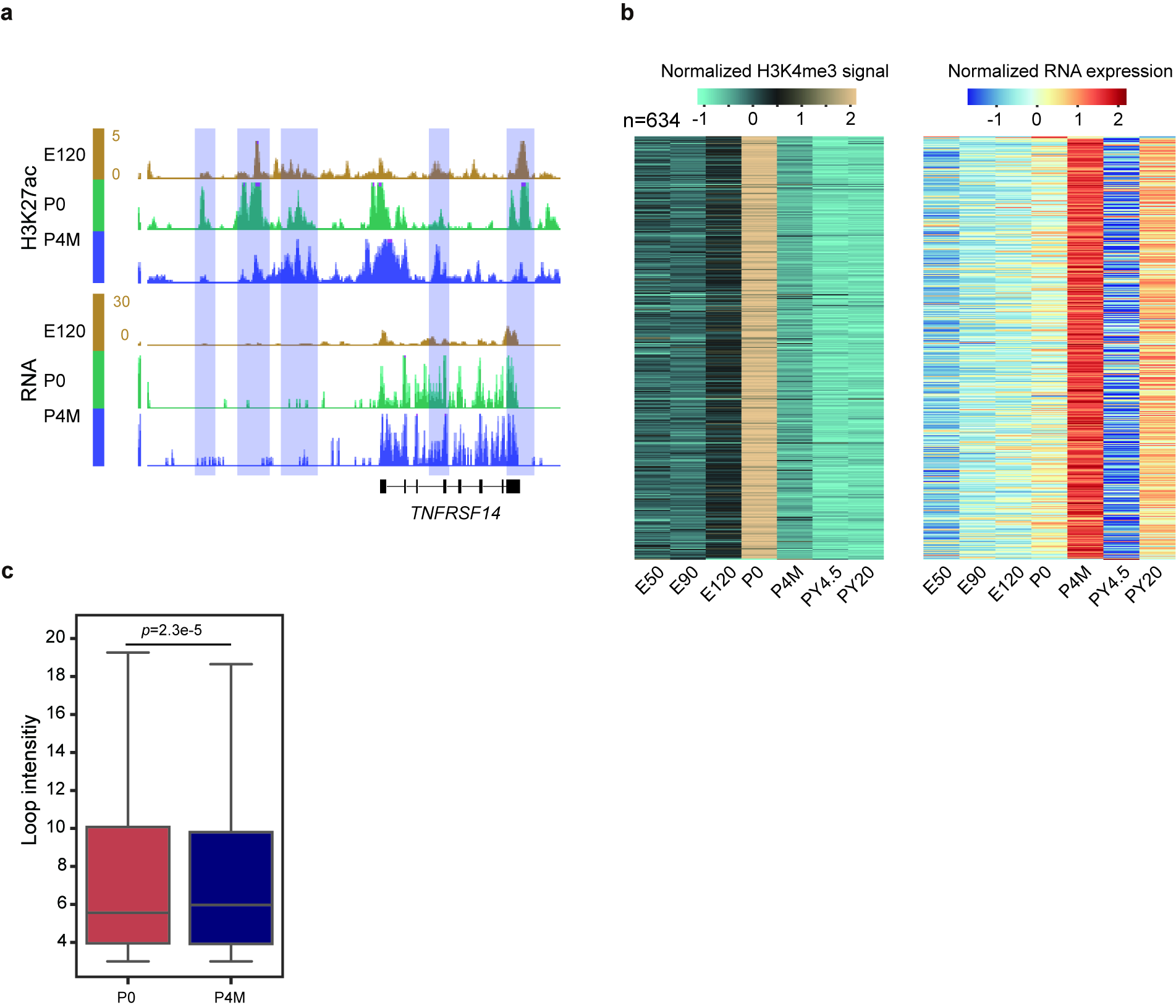


## Supplementary Figure 6. The influence of H3K27ac and H3K4me3 signal alterations on gene expression across birth stage.

**(a)** Genome browser view of H3K27ac signal and gene expression pattern at the *TNFRSF14* locus. Blue shadows highlight the *cis*-elements that exhibit changes in H3K27ac signal between the P0 and P4M stages. The expression levels of *TNFRSF14* are not significantly changed between the P0 and P4M stages. **(b)** Heatmap showing promoter H3K4me3 signal and RNA expression of a set of genes. The number of the genes is shown. These genes harbor H3K4me3 signal at the P0 stage. These genes are not or lowly expressed at the P0 stage, but highly expressed at the P4M stage. **(c)** Box plots comparing the loop intensity of the primed genes between the P0 and P4M stages. The primed genes are those shown in panel a. Wilcoxon rank sum test was used.


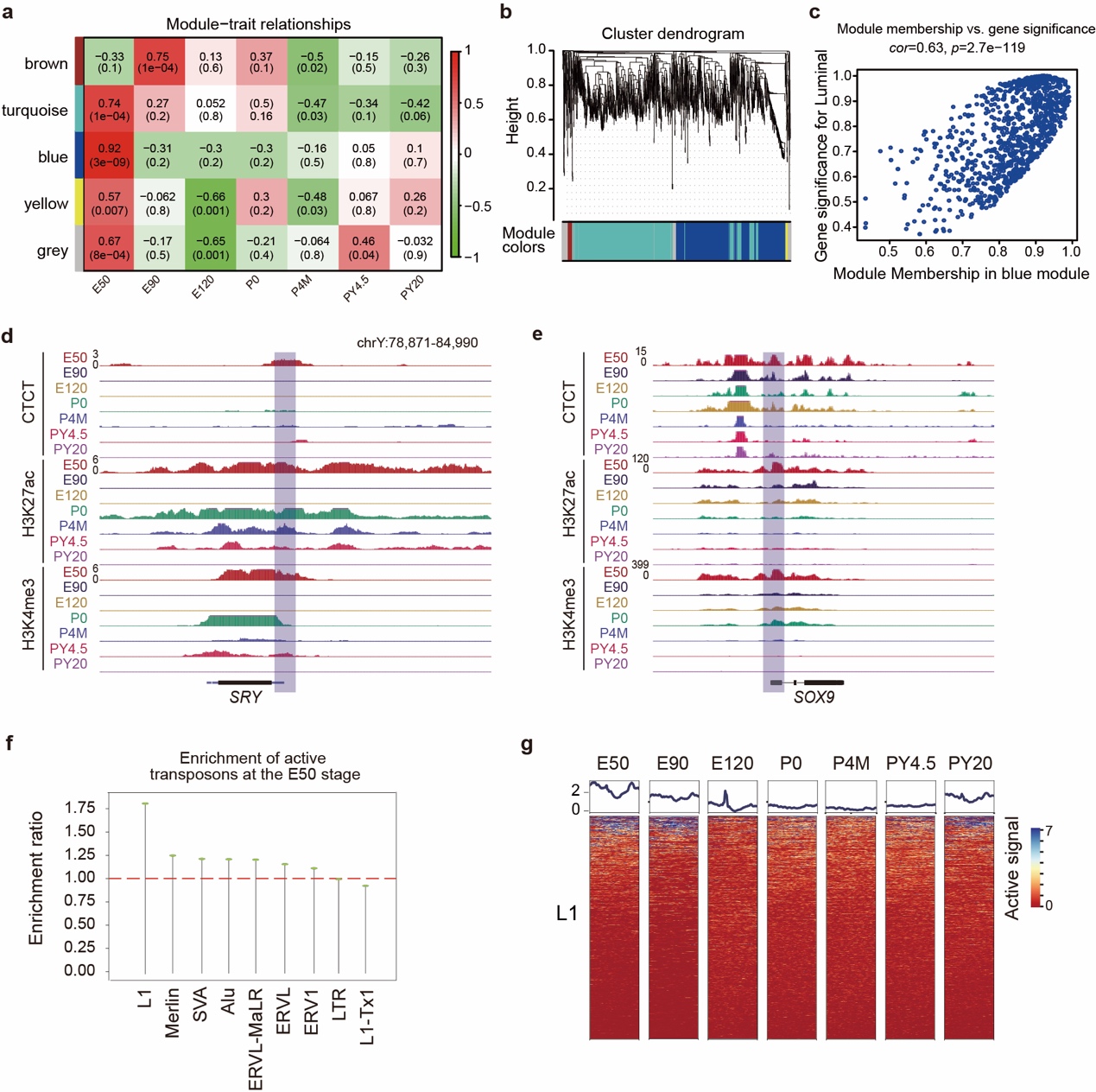


## Supplementary Figure 7. Regulatory modules of NPCs during neurogenesis.

**(a)** Heatmap showing the relationship between *cis*-element modules and stage traits. The corresponding correlation and *p*‐value are shown. Red color indicates positive correlation and blue color indicates negative correlation. **(b)** Hierarchical dendrogram of the *cis*-element modules. **(c)** Scatter plot showing the relationship between significance for active signal and module membership in blue module. **(d)** Genome browser view of CTCF, H3K27ac and H3K4me3 signal at *SRY* gene locus. The promoter region is highlighted in purple shadow. **(e)** Genome browser view of CTCF, H3K27ac and H3K4me3 signal at *SOX9* gene locus. The promoter region is highlighted in purple shadow. **(f)** Bar plot showing the enrichment of active signal in different classes of transposons at the E50 stage. **(g)** Meta plots and heatmaps showing the active signal of L1 during PFC development and ageing.


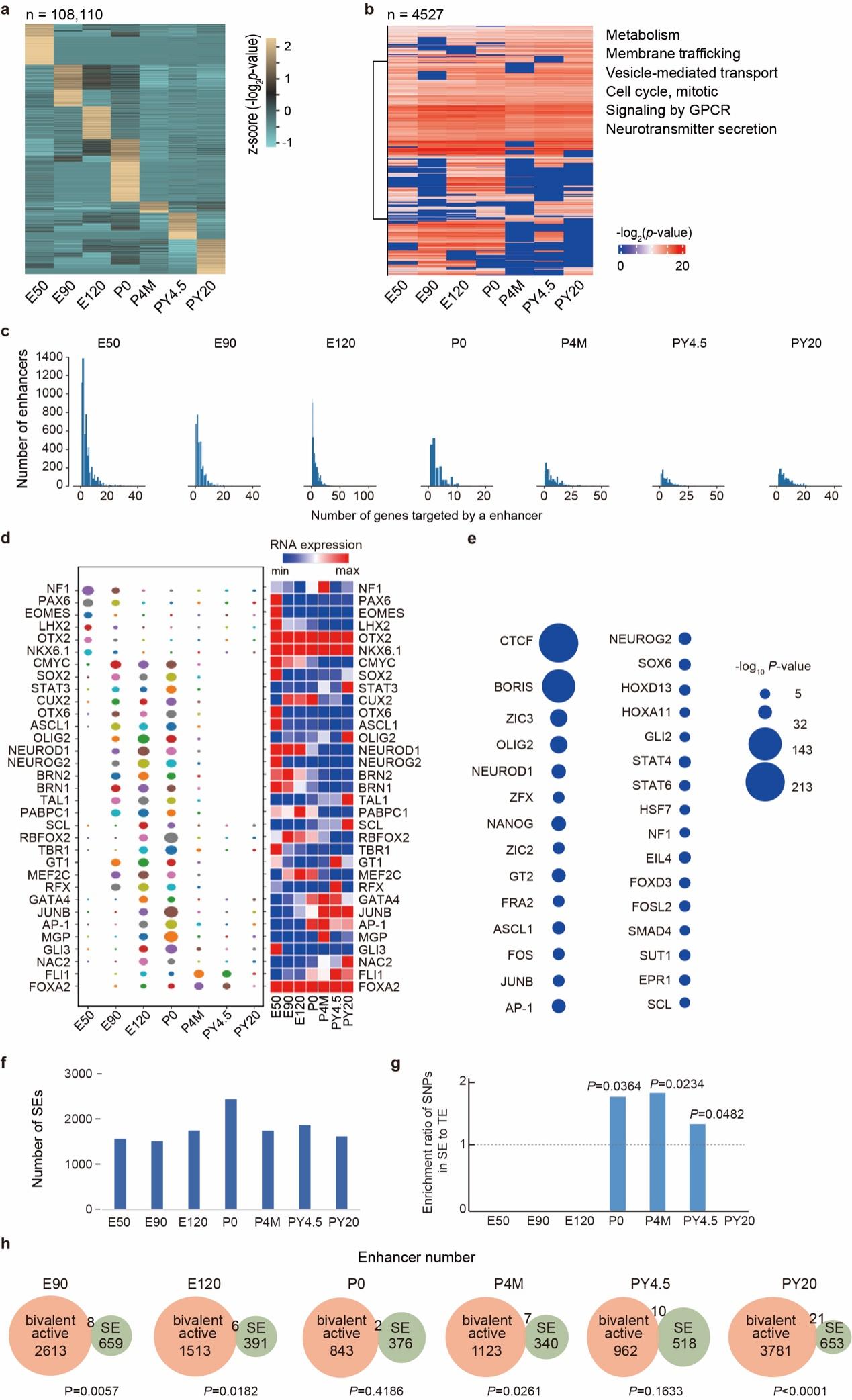


## Supplementary Figure 8. The dynamics of *cis*-element during PFC development.

**(a)** Heatmap showing H3K27ac signal of stage-specific putative enhancers. The number of the enhancers is shown. **(b)** Heatmap showing the H3K27ac signal of stage-common putative enhancers. The GO results of the genes associated with these putative enhancers are shown in the right. **(c)** Bar plots showing the numbers of enhancers associated with various numbers of genes in rhesus PFCs at different stages. **(d)** The enrichment of TF binding motifs in the stage-specific putative enhancers. The right heatmap showing the expression of these TFs. The sizes of dots in the left of the panel represent the enrichment score. **(e)** The enrichment of TF binding motifs in the stage-common putative enhancers. The sizes of dots in the left of the panel represent the enrichment score. **(f)** Bar plot showing the number of SEs in rhesus PFCs during development. **(g)** The enrichment ratios of intelligence-associated SNPs in SEs compared to TEs at different development stages. Fisher’s exact test was used. **(h)** Overlap between the genes regulated by bivalent promoters and the genes regulated by SEs. Fisher's Exact test was used.


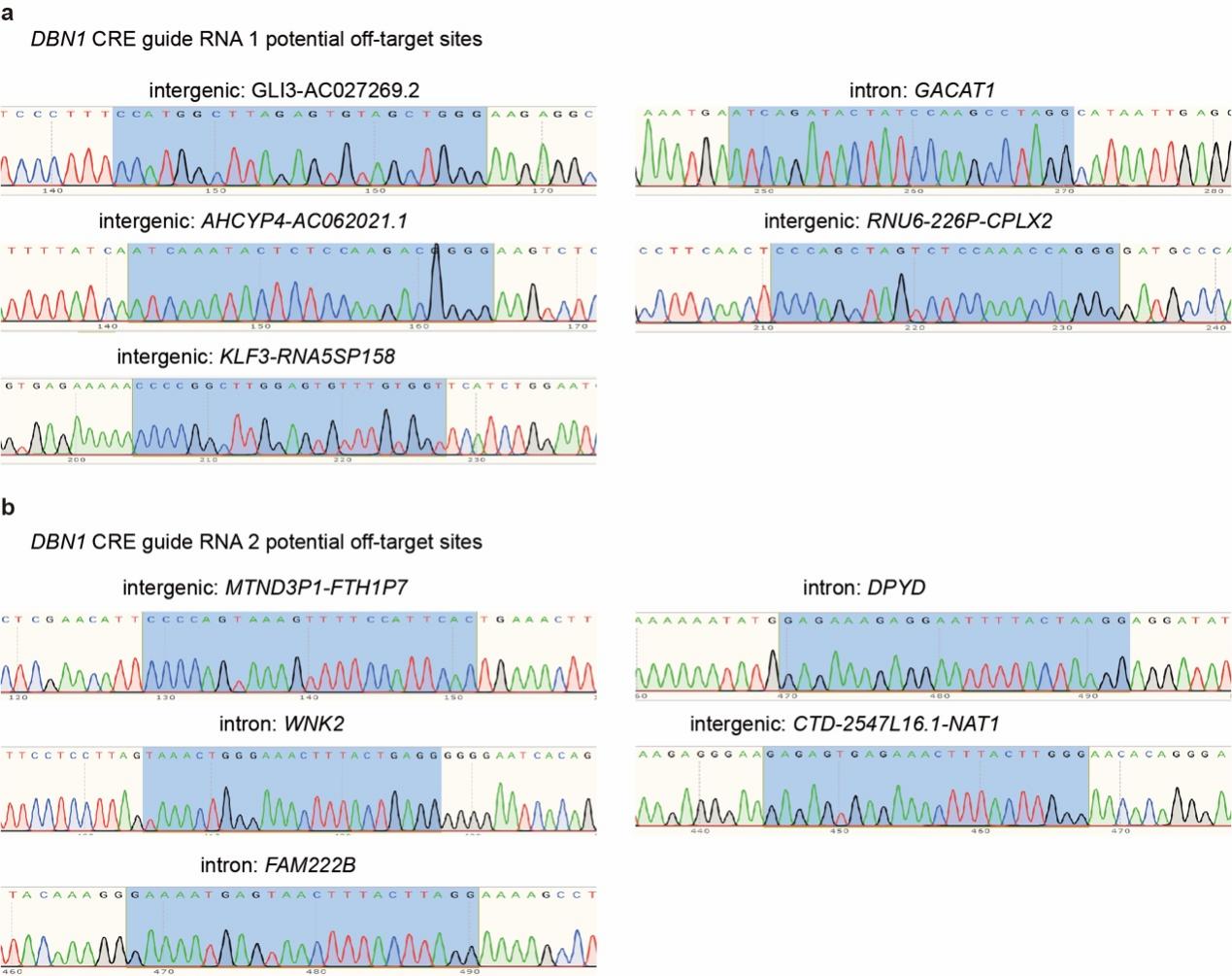


## Supplementary Figure 9. Evaluation of off-target effects of *DBN1* CRE sgRNAs

**(a-b)** Sanger sequencing results of potential off-target sites of *DBN1* CRE sgRNAs. For each sgRNA, the top 5 off-target sites were sequenced. All these potential off-target sites were not mutated. It indicates the specificity of sgRNAs is high.


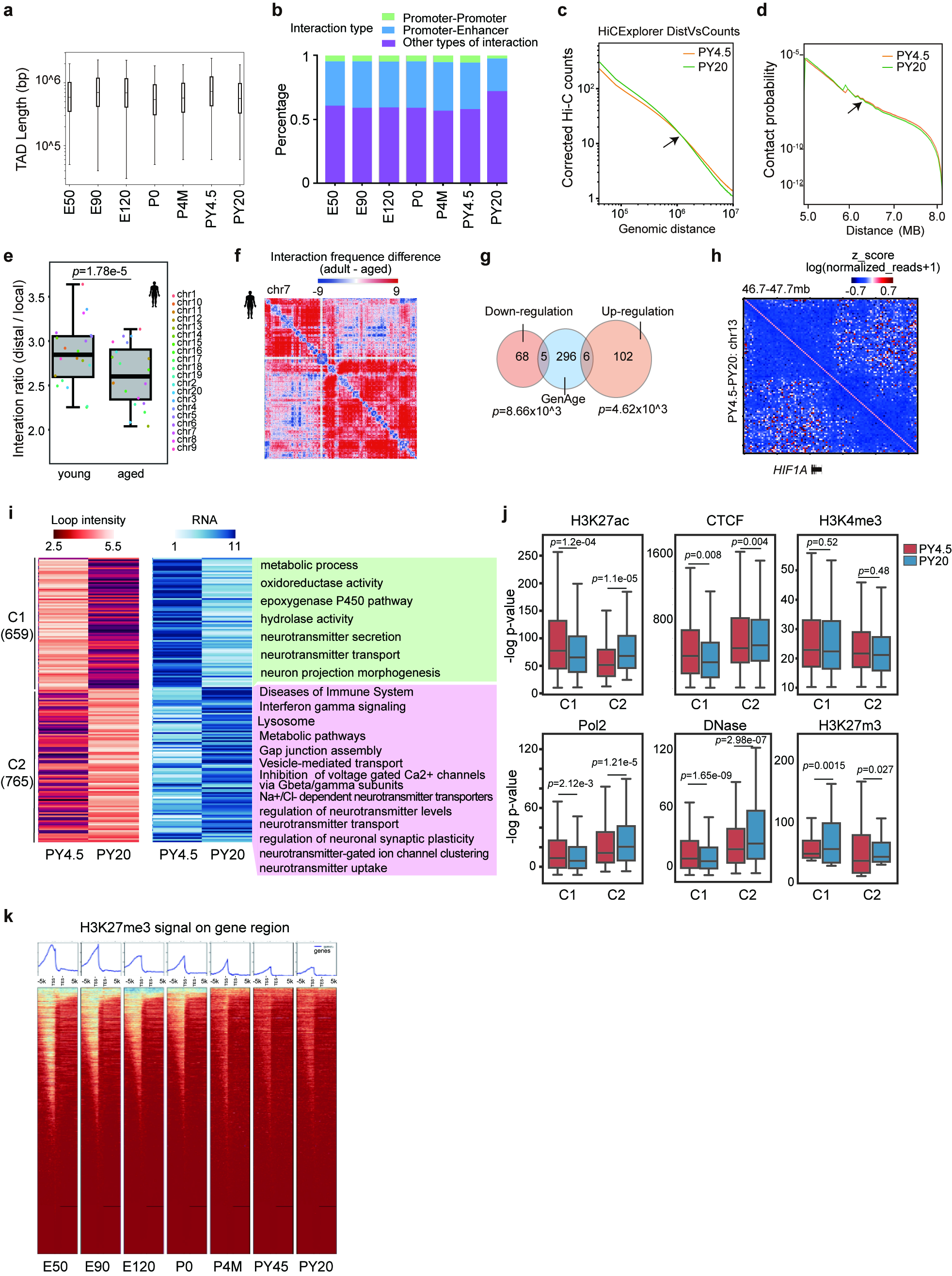


## Supplementary Figure 10. Chromatin interaction, H3K27me3 signal and gene expression are changed during PFC aging.

**(a)** Box plot showing the lengths of TADs in rhesus PFC samples. **(b)** The percentage of different types cis-element interactions at different developmental stages. **(c)** Distribution of Hi-C interaction counts between genomic bins with different genomic distances at the P4M and PY20 stages. Blue line represents the PY4.5 stage, red line represents the PY20 stage. (**d)** Contact probabilities between genomic bins with different ranges of genomic distances at the PY4.5 and PY20 stages. **(e)** Box and jitter plots showing the ratio of intra-TAD interaction to inter-TAD interaction in each chromosome in human between young and aged samples. Wilcoxon rank sum test was used. **(f)** Heatmap showing the interaction frequency difference between aged and young human brains. The values were calculated by subtracting the values of aged sample from the values of young sample. **(g)** Venn diagram showing the overlap between upregulated or downregulated genes in the split TAD boundaries and the genes in GenAge database. **(h)** Heatmap showing the interaction frequency difference between the PY4.5 and PY20 stages at the locus of *HIF1A*. The values were calculated by subtracting the values of PY20 sample from the values of PY4.5 sample. **(i)** Heatmap showing loop intensity and expression levels of the genes whose promoter exhibiting loss (C1) or gain (C2) loop interaction. The GO results of these genes are shown in the right. **(j)** Box plots comparing the changes of epigenetic or protein binding signals between PY4.5 and PY20 stages for the genes analyzed in panel i. Wilcoxon rank sum test was used. **(k)** Meta plots and heatmaps showing the signal of H3K27me3 across protein-coding genes in rhesus PFCs at different stages.

## Supplementary Table S1. The information of rhesus PFC samples.

## Supplementary Table S2. Summary of sequencing data.

## Supplementary Table S3. The epigenetic signals and expression levels of genes with stage-specific epigenetic signals.

## Supplementary Table S4. The list and GO results of the genes in the switched compartments.

## Supplementary Table S5. The expression levels of the genes associated with dynamic TAD boundaries between two adjacent stages.

## Supplementary Table S6. A consolidated marker gene list of human neural cell types.

## Supplementary Table S7. Consolidated gene lists of neuronal diseases.

## Supplementary Table S8. Consolidated list of genes or TFs associated with neuronal diseases that harboring active epigenetic signal in rhesus PFCs.

## Supplementary Table S9. The normalized DNA methylation and H3K27me3 levels of the genes exhibiting transition from H3K27me3 to DNA methylation.

## Supplementary Table S10. The gene ages of the genes associated with cis-element modules.

## Supplementary Table S11. The normalized active signal of super enhancers and expression levels of associated genes.

## Supplementary Table S12. TAD boundary tendencies and RNA expression levels of genes located in the newly formed TAD boundaries caused by the increased short-range interaction.

## Supplementary Table S13. Potential off-target sites of *DBN1* CRE sgRNAs and PCR primers for the amplification of these off-target sites.
